# Antibody Recognition of Different *Staphylococcus aureus* Wall Teichoic Acid Glycoforms

**DOI:** 10.1021/acscentsci.2c00125

**Published:** 2022-08-17

**Authors:** Cristina Di Carluccio, Pablo Soriano-Maldonado, Francesca Berni, Carla J. C. de Haas, A. Robin Temming, Astrid Hendriks, Sara Ali, Antonio Molinaro, Alba Silipo, Nina M. van Sorge, Mark J. van Raaij, Jeroen D. C. Codee, Roberta Marchetti

**Affiliations:** † Department of Chemical Sciences, University of Naples Federico II, Via Cinthia 4, 80126Naples, Italy; ‡ Departamento de Estructura de Macromoléculas, Centro Nacional de Biotecnología, Consejo Superior de Investigaciones Científicas (CNB-CSIC), Calle Darwin 3, 28049Madrid, Spain; § Leiden Institute of Chemistry, Leiden University, Einsteinweg 55, 2333 CCLeiden, The Netherlands; ∥ Medical Microbiology, UMC Utrecht, Utrecht University, 3508Utrecht, The Netherlands; ⊥ Department of Medical Microbiology and Infection Prevention, Amsterdam UMC, University of Amsterdam, 1105 AZAmsterdam, The Netherlands; # Netherlands Reference Laboratory for Bacterial Meningitis, Amsterdam UMC, 1105 AZAmsterdam, The Netherlands

## Abstract

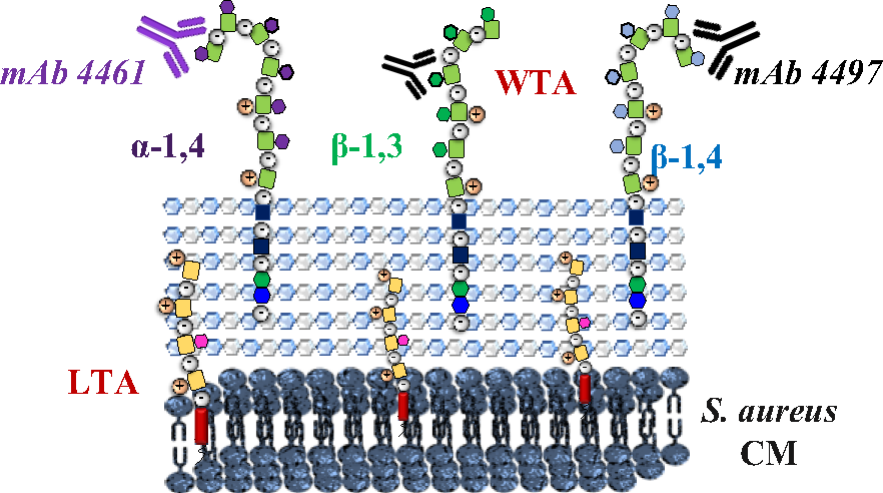

Wall teichoic acids (WTAs) are glycopolymers decorating the surface of Gram-positive bacteria and potential targets for antibody-mediated treatments against *Staphylococcus aureus*, including methicillin-resistant (MRSA) strains. Through a combination of glycan microarray, synthetic chemistry, crystallography, NMR, and computational studies, we unraveled the molecular and structural details of fully defined synthetic WTA fragments recognized by previously described monoclonal antibodies (mAbs 4461 and 4497). Our results unveiled the structural requirements for the discriminatory recognition of α- and β-GlcNAc-modified WTA glycoforms by the complementarity-determining regions (CDRs) of the heavy and light chains of the mAbs. Both mAbs interacted not only with the sugar moiety but also with the phosphate groups as well as residues in the ribitol phosphate (RboP) units of the WTA backbone, highlighting their significant role in ligand specificity. Using elongated WTA fragments, containing two sugar modifications, we also demonstrated that the internal carbohydrate moiety of α-GlcNAc-modified WTA is preferentially accommodated in the binding pocket of mAb 4461 with respect to the terminal moiety. Our results also explained the recently documented cross-reactivity of mAb 4497 for β-1,3/β-1,4-GlcNAc-modified WTA, revealing that the flexibility of the RboP backbone is crucial to allow positioning of both glycans in the antibody binding pocket.

## Abstract 2

Monoclonal antibody (mAb)−wall teichoic acid (WTA) interactions have been investigated to afford key information for the development of effective antibody therapeutics against *S. aureus* infections.

## Introduction

*Staphylococcus aureus* is a Gram-positive bacterium that asymptomatically colonizes 30% of the human population. However, it can also cause severe infections, including bacteremia, staphylococcal toxic shock syndrome, endocarditis, and osteomyelitis.^1,2^ The World Health Organization has ranked antibiotic-resistant *S. aureus* (e.g. methicillin-resistant *S. aureus*, MRSA) as a high-priority pathogen, as there is a growing health concern.^3^ Therefore, the development of new nonantibiotic therapeutic approaches is urgently needed.

**Figure 1:**
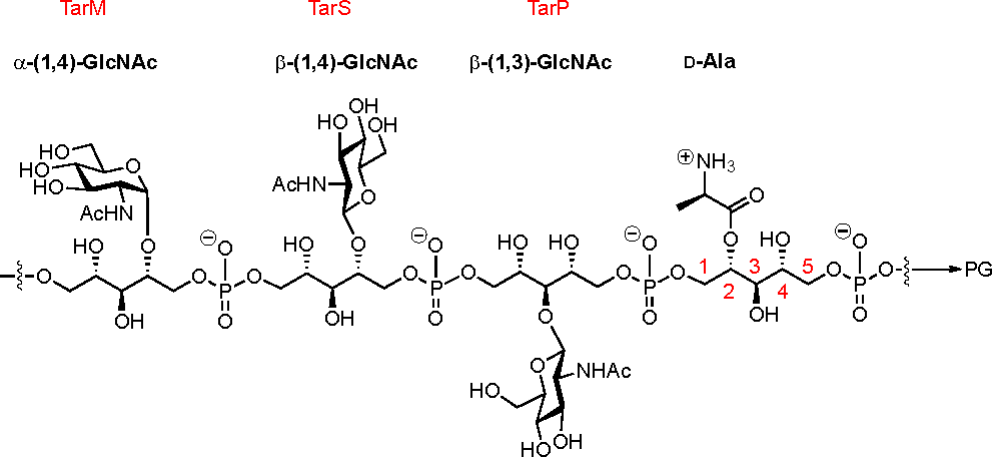
*S. aureus* WTA is composed of repeating ribitol-5-phosphate (RboP) residues that can be decorated on the C-2 position with d-alanine esters and *N*-acetyl-d-glucosamine (GlcNAc) residues. The biosynthesis enzymes TarS, TarM, and TarP decorate the RboP backbone at C4 with β-GlcNAc, at C4 with α-GlcNAc, and at C3 with β-GlcNAc, respectively, to create different WTA glycoforms.

The thick peptidoglycan layer that characterizes the cell wall of Gram-positive bacteria is densely conjugated with wall teichoic acids (WTAs).^4^ These linear anionic glycopolymers are crucial not only for bacterial physiology but also for interaction with the host through its contribution to colonization and pathogenesis.^5^ WTAs, which can represent up to half of the cell wall mass,^6^ are anchored to peptidoglycan and are composed of ribitolphosphate (RboP) repeating units.^7^*S. aureus* WTAs show limited structural variation among different *S. aureus* strains, which is influenced by gene expression and environmental conditions.^8^ First of all, the RboP subunits can be functionalized by the amino acid d-alanine, commonly linked to C2 of the RboP subunits through an ester linkage (see Figure 1). In addition, the WTA RboP backbone can be modified by the action of three dedicated glycosyltransferases, which attach *N*-acetylglucosamine (GlcNAc) moieties with different stereochemistries (α or β) to different positions of the RboP subunits, resulting in β-1,3- (TarP), β-1,4 (TarS), and α-1,4 (TarM) glycotypes.^9,10^ These modifications are differently recognized by both innate and adaptive immune components, thereby affecting the capacity of host-mediated immune detection and clearance.^11^ Furthermore, these WTA GlcNAc moieties represent antigenic epitopes, being promising targets for active^12^ and passive immunization strategies as well as antibiotic delivery^2^ through targeting with monoclonal antibodies (mAbs).^13,14^

**Figure 2:**
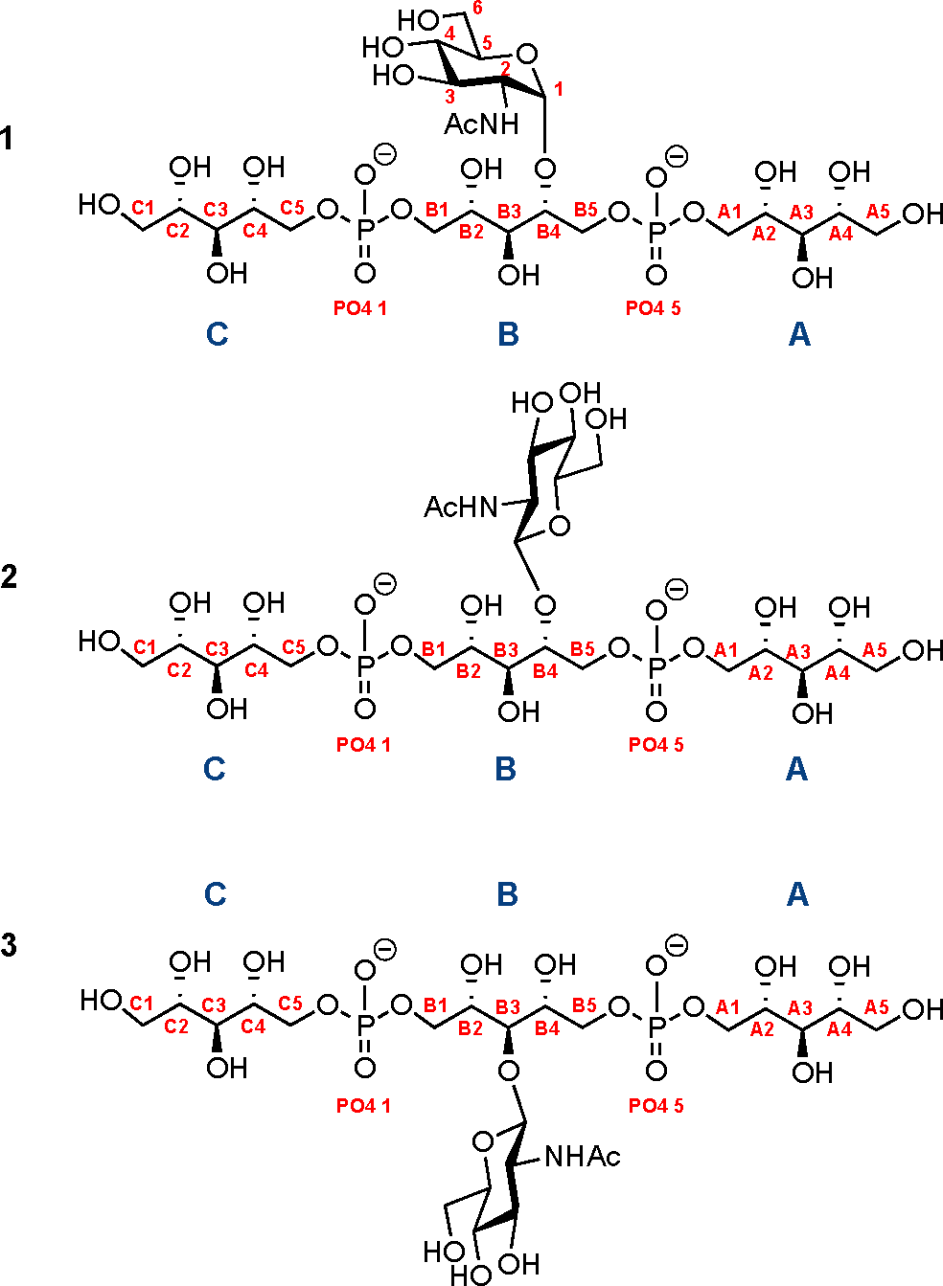
Ligands to probe antibody binding. The structures were designed to incorporate all structural features that were deemed important for mAb binding, a central glycosylated ribitol moiety flanked on each side by a RboP residue: (**1**) α-1,4-WTA; (**2**) β-1,4-WTA; (**3**) β-1,3-WTA.

Previous binding studies have shown the ability of patient-derived MRSA-targeting mAbs to recognize β-GlcNAc-modified WTA.^2,14^ Using “minimal epitopes” comprising a single RboP monomer with a single β-1,4-GlcNAc and a single phosphate group, it was revealed that both the carbohydrate and the phosphate group contribute to the binding with the mAb germline encoded heavy- and light-chain residues interacting with the GlcNAc.^2,14^ However, structural information regarding binding for mAbs targeting different glycoforms (β-1,3- and α-1,4GlcNAc) is currently lacking. Such studies require the use of well-defined synthetic fragments of these targets, which have only recently become available. We are now able to assemble all three WTA glycotypes through effective synthetic chemistry using (automated) DNA-synthesis techniques.^15^ The resulting library of synthetic WTA fragments consists of molecules that vary in RboP-chain length, glycotype (β-1,3-, β-1,4, and α-1,4), and the exact position and number of GlcNAcs on the RboP chain.^15^ Using this WTA library, we determined the fine specificities of four different WTA-specific mAbs, which were produced based on published sequences.^16^ The β-1,3-GlcNAc glycotype has been suggested to play a role in antibody immune evasion by *S. aureus*.^10^ However, we have previously shown, through the use of our well-defined WTA fragments, that β-1,3-GlcNAc-reactive antibodies are abundantly present in human serum.^17^ Moreover, β-1,4-GlcNAc mAbs are cross-reactive with β-1,3-GlcNAc WTA.^17^ We set out to determine the molecular basis for the cross-reactivity of β-1,4-/β-1,3-binding antibodies and pinpoint structural features that determine the recognition of α-1,4-GlcNAc WTA. To this end, we mapped the binding of two mAbs, i.e. the β-GlcNAc specific mAb 4497 and the anti-α-1,4-GlcNAc-WTA mAb 4461, using different well-defined synthetic WTA fragments. To unravel antibody–WTA binding at the atomic level, we performed WTA-microarray binding studies, X-ray crystallography, and NMR spectroscopy in combination with docking and molecular dynamic simulations. For crystallographic studies, we designed three WTA epitopes that consist of three ribitol residues carrying a single GlcNAc at the central ribose (WTA trimers **1**–**3**; see Figure 2). We reasoned that these structures would allow us to not only investigate the role of the GlcNAc in binding, as previously reported by Fong et al.,^14^ but also assess the contribution of the two flanking phosphates and ribitol moieties.

The structural investigation of the α-1,4-GlcNAc-specific mAb revealed how GlcNAc is positioned in the binding pocket of the antibody, with the RboP backbone making contact to the Ab surface, demonstrating that the nature of the backbone is critical in specificity. In addition, the internal α-GlcNAc residues are better recognized than the α-GlcNAc moieties at the terminal ribitol phosphate chains. For the β-GlcNAc-specific mAb 4497, we showed that the flexibility of the RboP backbone is crucial to accommodate both the β-1,3- and β-1,4-regioisomers.

## Results

### mAb-WTA Binding Studies Using Teichoic Acid (TA) Microarrays

**Figure 3:**
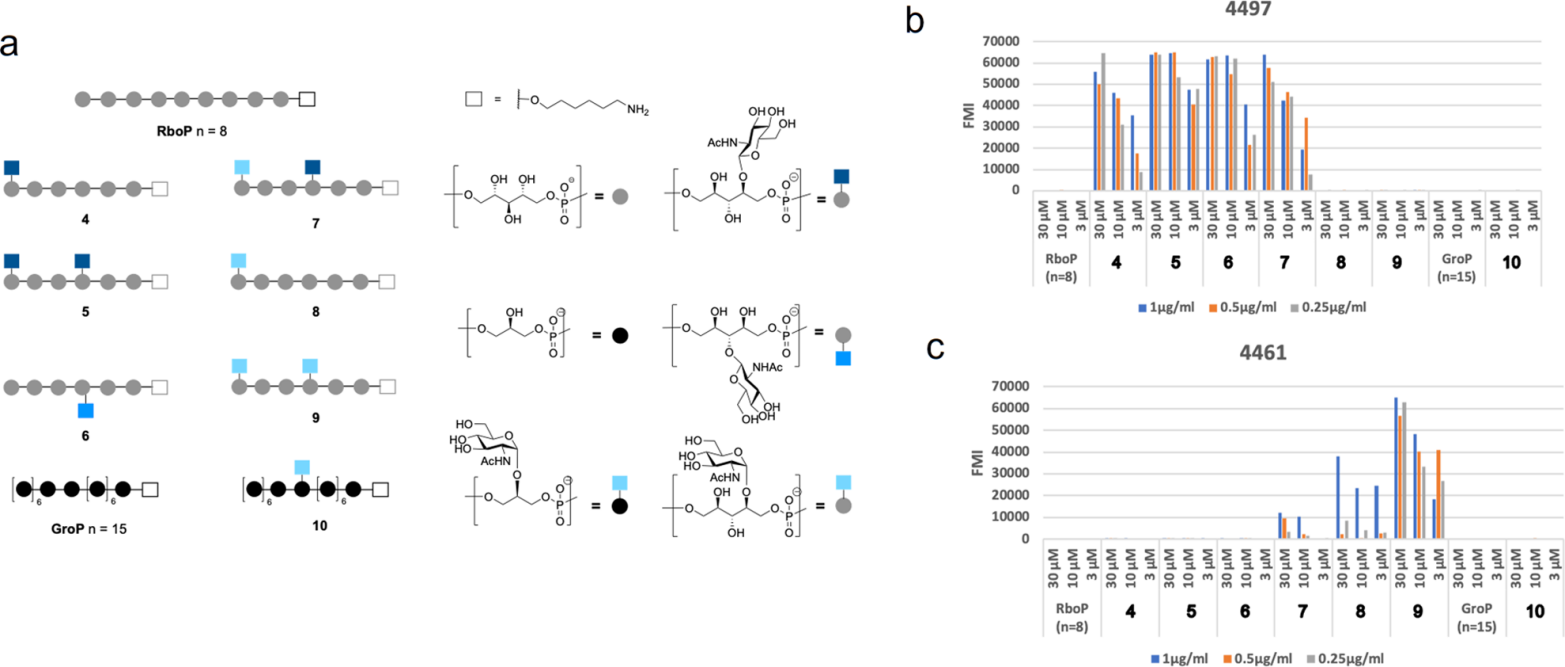
(a) Schematic overview of the TA microarray library. Microarray results for (b) mAb 4497 and (c) mAb 4461 at different concentrations. Legend: 1 μg/mL, blue; 0.5 μg/mL, orange; 0.25 μg/mL, gray. Values refer to the average of the fluorescent median intensity of three spots for each compound printed at 30, 10, and 3 μM concentrations. *n* = number of repeating units.

We recently developed a TA microarray to evaluate antibody binding to a large set of poly(glycerolphosphate) (GroP)-based lipoteichoic acid (LTA) oligomers.^18^ We have applied this TA microarray to map the recognition of different mAbs as well as polyclonal sera.^15,19,20^ To start our binding studies of the mAbs 4497 and 4461, we here generated an array presenting both WTA and LTA fragments (Figure 3).

Different WTA-glycotypes **4**–**9**, carrying none, one, or two GlcNAc-residues, were included in the array (Figure 3a). We also included a GroP-based pentadecamer (GroP, *n* = 15) and GroP oligomer **10**, featuring α-GlcNAc at the C-2 position of a glycerol unit. The compounds, equipped with an aminohexanol linker, were immobilized on an epoxide-functionalized glass slide in three different concentrations (30, 10, and 3 μM) in triplicate. Figure 3b,c shows the binding specificities of mAb 4497 and mAb 4461, respectively. In line with our previous reports, mAb 4497 recognized both the β-1,3- and β-1,4-GlcNAc glycosylated RboP hexamers **5** and **6**, and the presence of a single β-GlcNAc substituent was sufficient for recognition (as in WTAs **4** and **7**). mAb 4461 showed clear specificity for the RboP hexamers decorated with α-1,4-GlcNAc substituents (**7**–**9**). The array revealed that the mAbs interacts more strongly when α-GlcNAc is present in the middle of the RboP chain (as in WTA **9**) in comparison to binding when the α-GlcNAc is positioned at the terminal RboP residues (in **7** and **8**). Notably, GroP-based TA **10** was not recognized, indicating that the RboP backbone plays a crucial role in antibody binding.

### IgG 4461 Binding of α1,4-GlcNAc WTA Fragments

#### mAb 4461–(α-1,4)-GlcNAc WTA-Trimer **1**: Crystallographic Data

**Figure 4:**
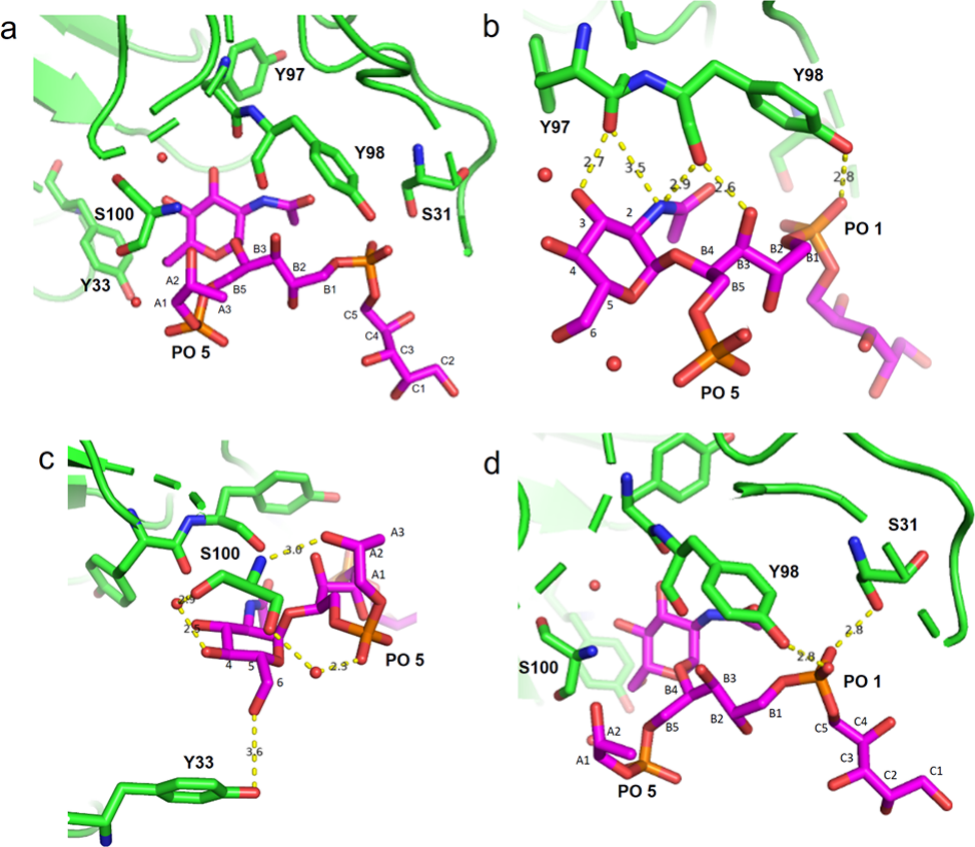
Different views of the binding site of IgG mAb 4461 in complex with (α-1,4)-GlcNAc WTA (ligand 1). (a) The α-GlcNAc-modified WTA **1** is bound in a cavity formed between the CDR of the heavy and light chains of the antibody. Residues S31, Y97, Y98, S100, and Y33 are involved in H-bond interactions with the ligand. (b) Residues Y97 and Y98 establish H-bonds with the GlcNAc ring, with the phosphate group **1**, and with the Rbo-B3 OH. (c) Residue S100 forms a H-bond with the Rbo-A2 OH as well as the GlcNAc C4-OH and phosphate group 5 through a water molecule. Residue Y33 interacts *via* an H-bond with GlcNAc C6-OH. (d) Residues S31 and Y98 establish H-bonds with an oxygen atom of phosphate group 1. The ligand (α-1,4)-GlcNAc WTA **1** is shown in magenta.

We solved the crystal structure of IgG mAb 4461 in a complex with the (α-1,4) ligand **1** at 1.45 Å resolution (PDB code 7QXC). mAb 4461 interacted with the α-GlcNAc-modified WTA in a cavity formed between the complementarity-determining region (CDR) of the heavy and light chains of the antibody (Figure 4a).

The structure also revealed that both phosphate groups are involved in ligand binding. This provides an explanation for the observation with the TA microarray that this mAb binds more strongly to internal GlcAc residues, being flanked by two phosphates, than to GlcNAc appendages of a terminal RboP moiety, having only one neighboring phosphate. The crystal structure revealed the amino acid residues of both the light and heavy chains involved in binding WTA trimer **1**. The carbonyl group of the light-chain residue Y98 was involved in a H-bond with the amide of the GlcNAc at 2.9 Å and with the Rbo-B hydroxyl group 3 at 2.6 Å, while its hydroxyl group made a H-bond with an oxygen atom (not involved in the phosphodiester linkage) of phosphate 1 at 2.8 Å. The carbonyl group of Y97 established two H-bonds, with the GlcNAc C3 hydroxyl group at 2.7 Å and with the GlcNAc acetamide at 3.5 Å (Figure 4b). S100 could form a H-bond with the GlcNAc C4-OH and the phosphate group 5 through two water molecules at 2.9 and 2.7 Å, respectively. This residue also formed a third H-bond with the Rbo-A 2 hydroxyl group at 3 Å (Figure 4c). Although the external Rbo units of the ligand are flexible and Rbo-A is therefore not completely defined in the crystal structure, we observed that RboA as well as phosphate groups 1 and 5 were involved in ligand interactions. The heavy-chain residue Y33 interacted with the WTA ligand through the hydroxyl group, which could establish a H-bond with the GlcNAc C6-OH at 3.6 Å (Figure 4c). Finally, residue S31 established a H-bond with an oxygen of phosphate 1 at 2.8 Å (Figure 4d). The interactions revealed by the crystallographic structure also provided insight into why the α-GlcNAc attached to the GroP backbone is not recognized by the antibody. This LTA ligand lacks the interactions of the properly positioned phosphate groups. In addition it likely experiences repulsive steric and charge interactions, for example between the glycerol phosphate and the amide of Y98, which makes a positive contribution to the binding of the RboP ligand **1**.

#### mAb 4461–(α-1,4)-GlcNAc WTA-Trimer **1**: NMR and MD Data

**Figure 5:**
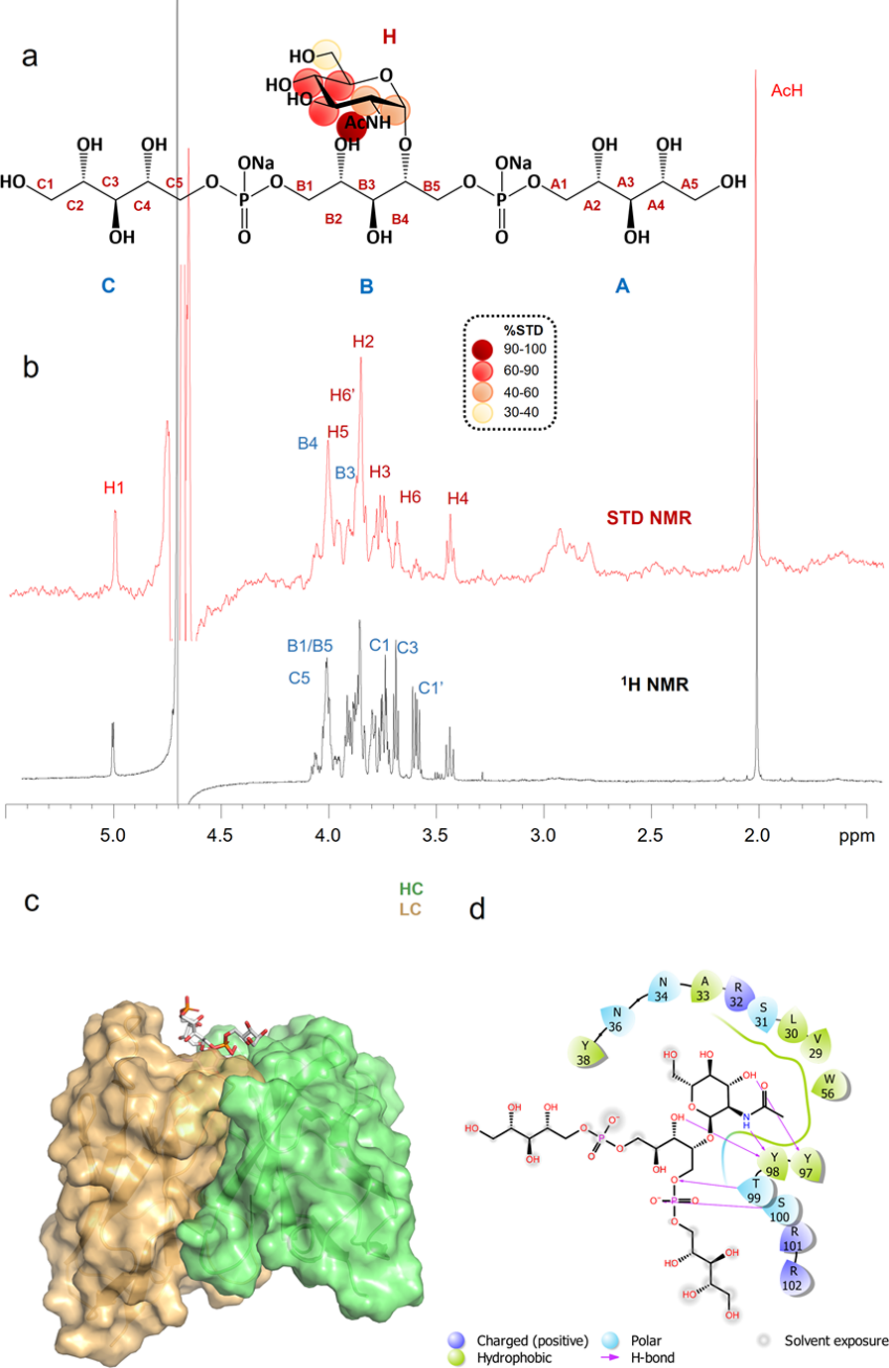
STD NMR and MD results of mAb 4461 and (α-1,4)-GlcNAc WTA, ligand 1 binding. (a) Epitope map of **1** interacting with mAb 4461 (only protons exhibiting %STD above 30% are indicated in the epitope mapping). (b) STD-NMR spectrum (red) of IgG 4461–**1** and the unsaturated reference spectrum (black). (c) 3D view of the mAb4461–**1** complex obtained from a molecular dynamics simulation. (d) Diagram of the interactions between mAb 4461 and **1** resulting from the best pose of the MD: solid arrows represent hydrogen bonds with functional groups of the amino acids of the antibody; the other residues in the binding pocket participate in polar and hydrophobic interactions.

The interactions between mAb 4461 and the synthetic glycosylated WTA oligomers were next explored in solution by NMR spectroscopy (see Table S2). To this end, STD NMR experiments were first performed on WTA-trimer **1** (Figure 5), allowing us to map positions involved in binding.^21^

As inferred from the intense STD signals of the sugar protons, the carbohydrate ring strongly contributed to the mAb interaction, while lower STD effects were observed for the ribitol chain residues (Figure 5a,b). To estimate the contacts of the ligand protons with the mAb 4461 antibody surface, the highest STD enhancement, observed for the acetyl group of GlcNAc, was set to 100% and all the other STD percentages were normalized accordingly (Figure 5b). The GlcNAc H3, H4, and H5 showed %STD values between 60% and 90%, indicating significant contribution to the binding. The magnetization was also transferred from the antibody to the GlcNAc H2 and to the anomeric proton, resulting in STD effects in the range of 40–60%. Lower STD responses were obtained for the GlcNAc H6 protons, indicating that this group associated in a weaker fashion with the antibody surface. Finally, lower STD signals, around 20–30%, attributed to the portion of the “internal” ribitol phosphate unit B and to some protons of the terminal Rbo chains, were also observed. Overall, the STD NMR analysis in solution confirmed the importance of the GlcNAc moiety and the involvement of the RboP backbone in the interaction with mAb 4461.

Molecular dynamics simulations performed on the mAb 4461–WTA-trimer **1** complex permitted further investigation of the recognition of the (α-1,4)-GlcNAc-modified WTA. The MD simulation corroborated the results achieved by crystallographic and NMR data, confirming the strong contribution of the GlcNAc, inserted in the cavity formed between the CDRs of the heavy and light chains of the antibody, to mAb binding. Interactions between the Y97 and Y98 residues of the light chain and C3-OH and the acetamide group of GlcNAc were observed along the simulation. The MD data also validated the interaction of phosphate 5 with S100, and a H-bond between this phosphate group and T99 was also detected. This latter interaction cannot be seen in the crystallographic structure. Moreover, MD established transitory interactions of the phosphate group 1 with Y98 and S31 and, differently from X-ray analysis, also with R32 (see Figure S1). In accordance with the STD NMR and crystallographic data, the MD simulation allowed the identification of an interaction between the C3-OH of the Rbo-B chain with Y98. Finally, despite the flexibility of the Rbo A and C arms, interactions of the C unit and the antibody could be observed. The A unit was more solvent exposed. In the crystallographic structure this chain was poorly defined, indicating its significant freedom of motion also *in crystallo*.

#### mAb 4461–(α-1,4)-GlcNAc WTA-Hexamer **9**

**Figure 6:**
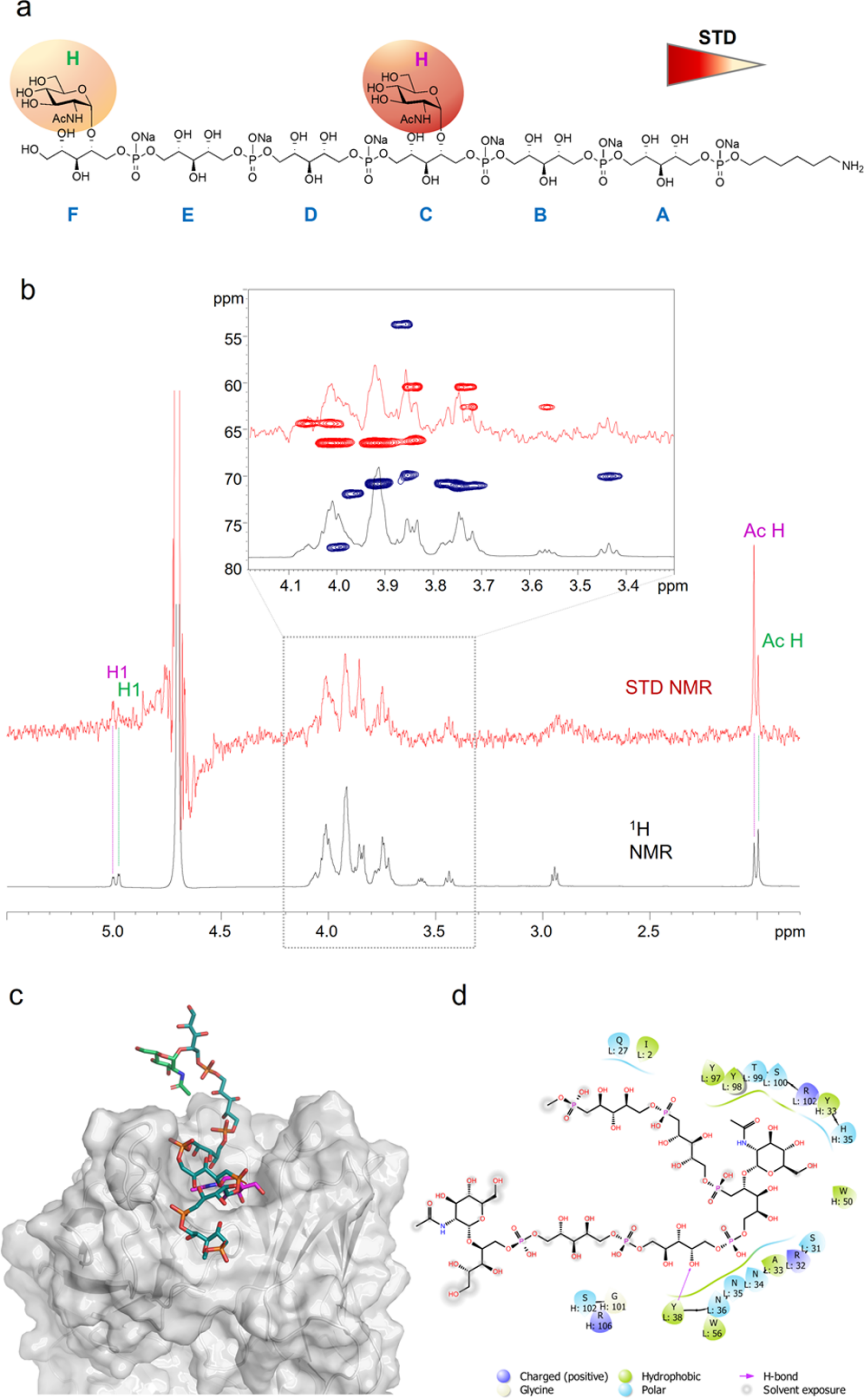
STD NMR and MD results of mAb 4461 and WTA hexamer **9** binding. (a) Epitope map of 9 interacting with IgG 4461. (b) STD-NMR spectrum (red) of mAb 4461–**9** and the unsaturated reference spectrum (black). (c) 3D view of the mAb 4461–**9** complex: the “internal” residue into the antibody binding site is shown in purple, and the “terminal” GlcNAc is shown in green. (d) Diagram of the interactions between mAb 4461 and **9** resulting from the manual docking: solid arrows represent hydrogen bonds with functional groups of the amino acids of the antibody; the other residues in the binding pocket participate in polar and hydrophobic interactions.

The interaction of mAb 4461 with WTA hexamer **9**, containing two α-GlcNAc units, was investigated next (Figure 6).

The STD NMR spectra revealed that the RboP monomers carrying the GlcNAc moieties (the C and F units) interacted differently with the antibody than the RboP monomers that were not glycosylated, displaying different STD enhancements. The STD NMR data further revealed that the “internal” GlcNAc contributed more to the binding than the “terminal” sugar (Figure 6a), corroborating the microarray results (Figure 3). This is more clear from a comparison of the two STD signals belonging to the acetyl groups of the “terminal” and “internal” GlcNAc residues, indicated in green and purple, respectively, in Figure 6a,b,c. Indeed, the acetyl group of the internal GlcNAc gave rise to the highest STD effect, set to 100% (Figure 6a, b), while the terminal GlcNAc showed less than half of the %STD value. Accordingly, the proton H1 of the internal GlcNAc residue (purple in Figure 6) revealed a major STD contribution, while the STD signal of the anomeric proton of the terminal sugar ring (green in Figure 6) was significantly lower.

A 3D view of ligand binding to mAb 4461 was achieved by manually docking the ligand to the mAb as depicted in Figure 6c. The internal sugar moiety (depicted in purple) was inserted in the antigen-binding site and provided the main interactions with mAb 4461, together with the RboP C and D units. In contrast, the terminal RboP moieties protruded away from the binding site. The 2D diagram of interactions (Figure 6d) shows the internal sugar embedded in the binding site of the mAb together with the protons of the neighboring Rbo chains; the terminal sugar is instead far away from the binding pocket, with the Rbo units E and F being solvent-exposed.

### Binding of mAb 4497 to β-GlcNAc-Modified WTA Fragments

#### mAb 4497–(β-1,4)-GlcNAc WTA-Trimer **2**: Crystallographic Data

**Figure 7:**
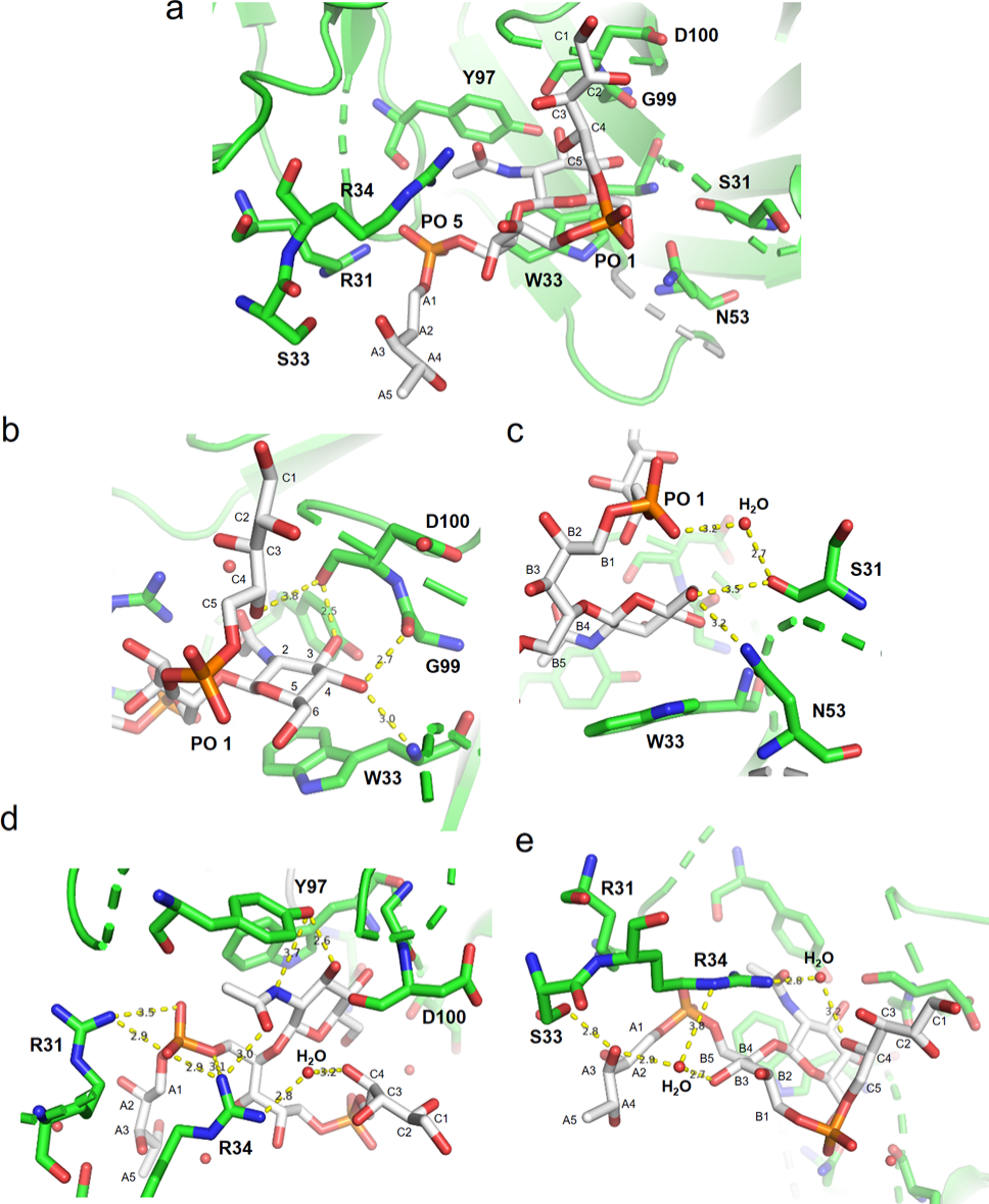
Different views of the binding site of IgG mAb 4497 in complex with ligand (β-1,4)-GlcNAc WTA ligand **2**. (a) The β-GlcNAc-modified WTA is located in a cavity formed between the CDR of the heavy and light chains of the antibody. (b) Residues W33, D100, and G99 are involved in H-bond interactions with GlcNAc. Residue W33 stacks with the ligand and establishes a H-bond with the GlcNAc C4-OH. Residue G99 forms a H-bond with GlcNAc-C4-OH while D100 makes H-bonds with GlcNAc C3-OH and the Rbo-C4 hydroxyl group. (c) Residues N53 and S31 form H-bonds with GlcNAc C6-OH and phosphate group 1 *via* a water molecule. (d) Residue Y97 interacts with the GlcNAc C3-OH and the acetamide group while residue R31 interacts with phosphate group 5. (e) R34 establishes H-bonds with two oxygen atoms of phosphate group 5, with the Rbo-C4, Rbo-B3, and Rbo-A3 hydroxyl groups through two water molecules, and with the GlcNAc acetamide group. Finally, residue S33 forms a H-bond with Rbo-A3. Ligand **2** (β-1,4) is shown in white.

We solved the structure of IgG mAb 4497 in a complex with (β-1,4)-ligand **2** at 1.65 Å resolution (PDB code 7QXD). The CDRs of the heavy and light chains of mAb 4497 form a cavity in which the β-GlcNAc-modified WTA is bound (Figure 7a), as previously described by Fong et al.^14^

Interestingly, our structure showed that, besides the GlcNAc, both phosphate groups and the Rbo-C and Rbo-A units were involved in the interaction with the mAb. The GlcNAc contributed to mAb binding through a stacking interaction with residue W33 from the heavy chain. An extended network of H-bonds further stabilized the sugar ring within the antibody (Figure 7d,e). A comparison between our crystal structure with those previously reported by Fong et al.^14^ (PDB codes: 6DWA, 4497/5P-β-(1–4)-GlcNAc-RboP; 5D6C, 4497/1P-β-(1–4)-GlcNAc-RboP), in which the same antibody was cocrystallized with a β-(1–4)-Glc-RboP ligand containing a single RboP moiety, indicated that nearly all the interactions involving GlcNAc were similar. The main difference was that in the previously reported structure the carbonyl group of D100 was pointing upward and thus could not make a H-bond with GlcNAc C3-OH, as was observed in our solved structure. The minimal ligands used in the previous study contained one ribose moiety and a single phosphate monoester, as opposed to the phosphodiesters present in the naturally occurring WTA and our ligands. Interestingly, whereas the network interactions in 5D6C matched quite well with those observed in our structure, with PO 5 interacting with R31 and R34 residues, the ribose moiety in 6DWA was placed quite differently because of the different position of the phosphate group in the RboP unit. This resulted in a different orientation within the binding pocket. We did not see the triangulation of the two arginine residues, which was shown to be crucial for the binding of 4497 with the β-1,4-GlcNAc WTA phosphomonoester, as reported by Fong et al.

#### mAb 4497–(β1,4)-GlcNAc WTA-Trimer **2**: NMR and MD Data

**Figure 8:**
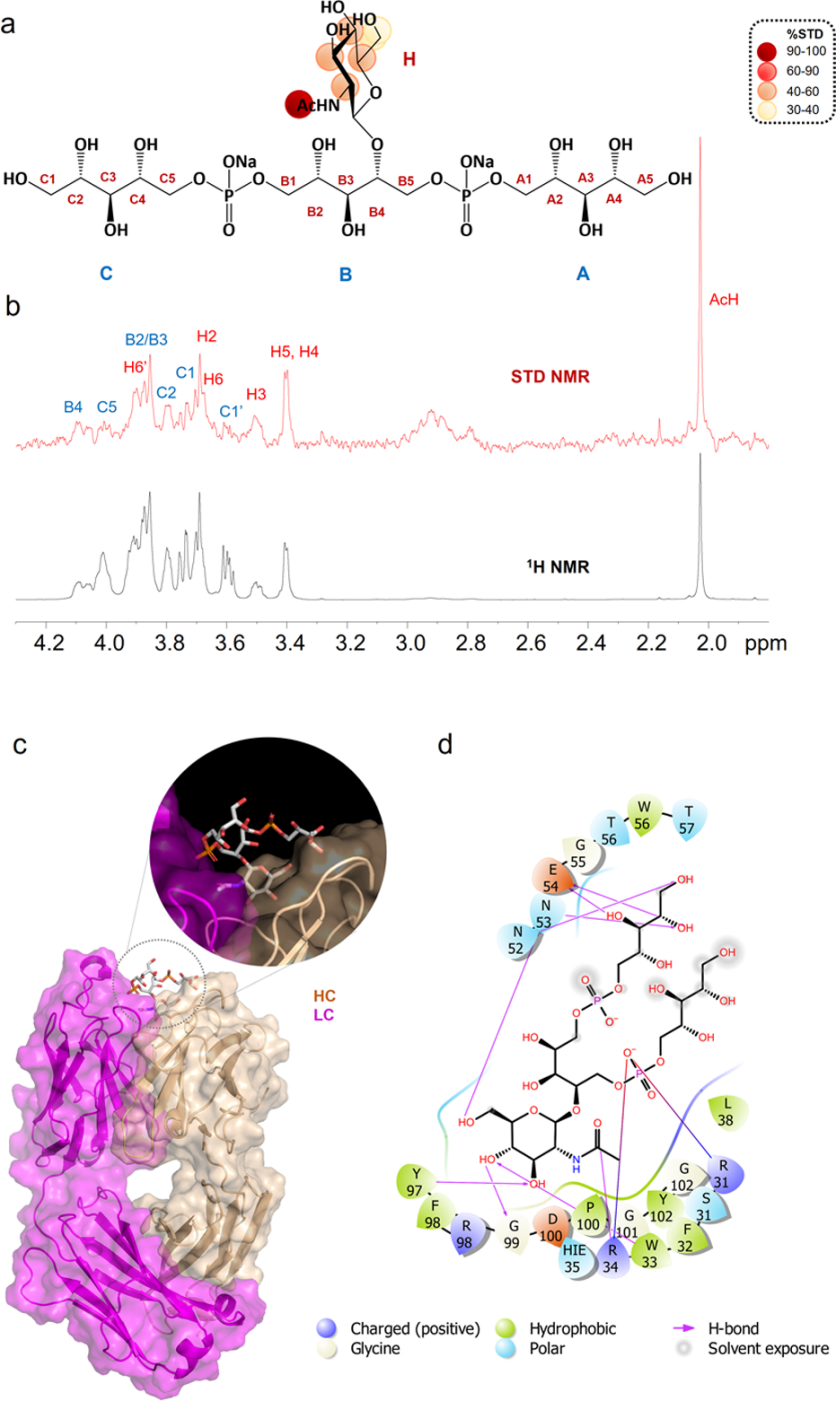
STD NMR and MD analysis of mAb 4497 and WTA-trimer **2** binding. (a) Epitope map of **2** interacting with IgG 4497 (only protons exhibiting %STD values above 30% are indicated in the epitope mapping). (b) STD-NMR spectrum (red) of mAb 4497–**2** and the unsaturated reference spectrum (black). (c) 3D view of the mAb 4497–**2** complex obtained from an MD simulation. (d) Description of the interactions between IgG 4497 and **2** resulting from a representative pose obtained by an MD simulation: solid arrows represent hydrogen bonds with functional groups of the amino acids of the antibody; the other residues in the binding pocket participate in polar and hydrophobic interactions.

The binding profile of WTA-trimer **2** bound to mAb 4497 was further examined by STD NMR spectroscopy (Figure 8a,b and Table S3), confirming a strong binding of the sugar with the antibody, with a weaker contribution from the RboP backbone.

The highest STD enhancements were observed for the GlcNAc acetyl group (set to 100%) and for protons H2–H5 of the sugar ring (around 50%), while protons at position 6, as well as those of the RboP B unit, showed lower STD effects. Finally, weak STD signals were observed for the protons of the Rbo unit C, as was further confirmed by MD data.

A representative pose of the complex mAb 4497–WTA trimer **2** obtained from 100 ns MD simulations is depicted in Figure 8c. Our MD results confirmed the interactions observed in the crystal structure (Figure 8d). In particular, stable hydrogen bonds, maintained for more than 90% of the simulation time, were monitored between G99 and the GlcNAc C4-OH and the hydroxyl group of Y97 with the GlcNAc C3-OH. Simultaneous contacts between phosphate group 5 with the guanidinium groups of R31 and R34 were established in the simulation. Furthermore, MD revealed additional interactions with respect to X-ray analysis. In detail, contacts between E54 with protons of the Rbo chain C, the C2 and C3-hydroxyls, and, to a lesser extent, N52 and N53 with the C1 and C2 hydroxyls of the same ribitol residue were found, indicating that this arm participates in antibody recognition, whereas the other RboP A arm was solvent-exposed.

#### mAb 4497–(β-1,3)-GlcNAc WTA-Trimer **3**: Crystallographic Data

Finally, the crystal structure of IgG mAb 4497 in complex with (β-1,3)-WTA, ligand **3**, was solved at a 1.84 Å resolution (PDB code 7QXE). The results revealed that the residues involved in the binding generally correspond to those observed for ligand **2** in complex with the same antibody.

**Figure 9:**
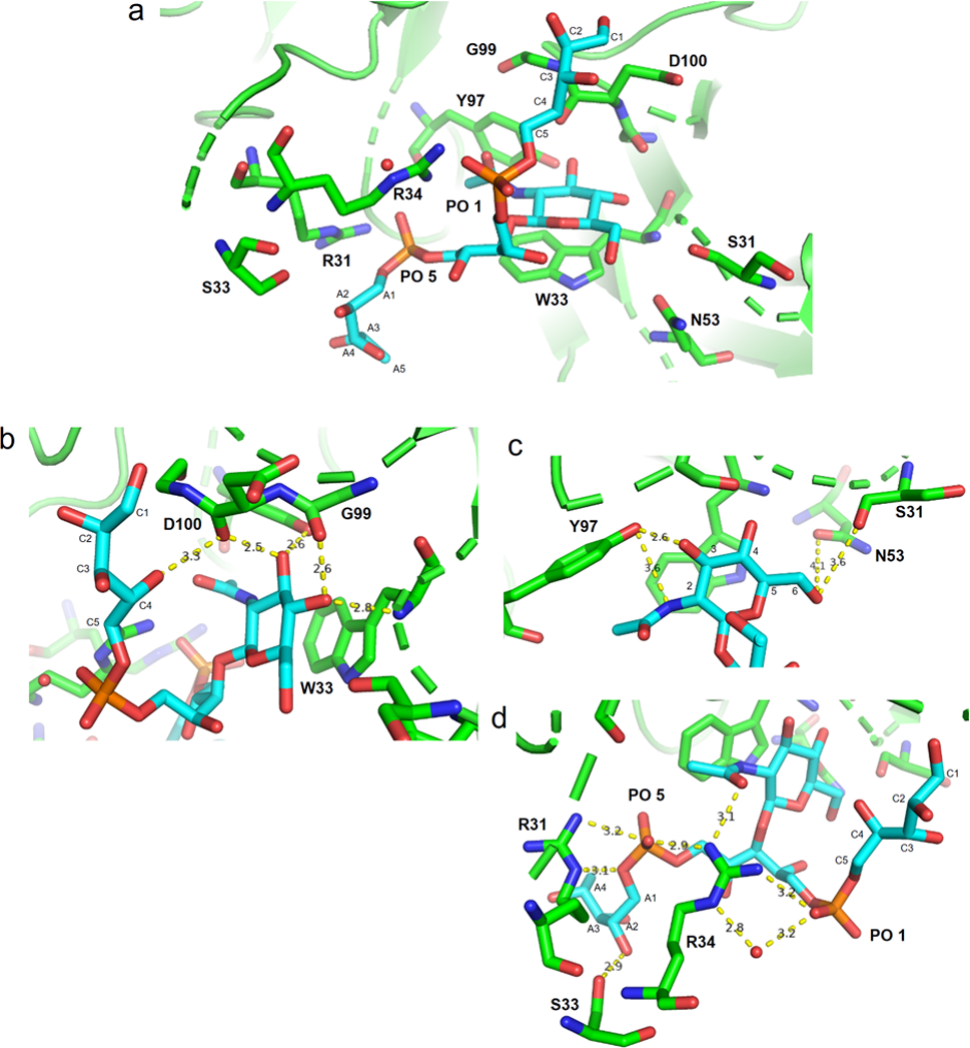
Different views of the binding site of IgG mAb 4497 in complex with (β-1,3)-ligand **3**. (a) The β-GlcNAc-modified WTA is located in a cavity formed between the CDR of the heavy and light chains of the antibody. (b) Residues D100, Y97, G99, S31, and W33 are involved in H-bond interactions with the GlcNAc. Residue W33 stacks with the GlcNAc and forms a H-bond with GlcNAc C4-OH. Residues G99, D100, and Y97 interact with the GlcNAc C4-OH and C3-OH, respectively. Residue D100 interacts with the Rbo-C4 hydroxyl group. (c) N53 is 4.1 Å from the GlcNAc C6-OH, while residue S31 interacts via H-bonds with GlcNAc C6-OH. Residue Y97 establishes a H-bond with the GlcNAc acetamide group and C3-OH. (d) R31 and R34 form H-bonds with both phosphate groups (1 and 5) and the acetamide group of GlcNAc. Finally, residue S33 establishes an H-bond with Rbo-A2. Ligand (β-1,3)-ligand **3** is shown in cyan.

**Figure 10:**
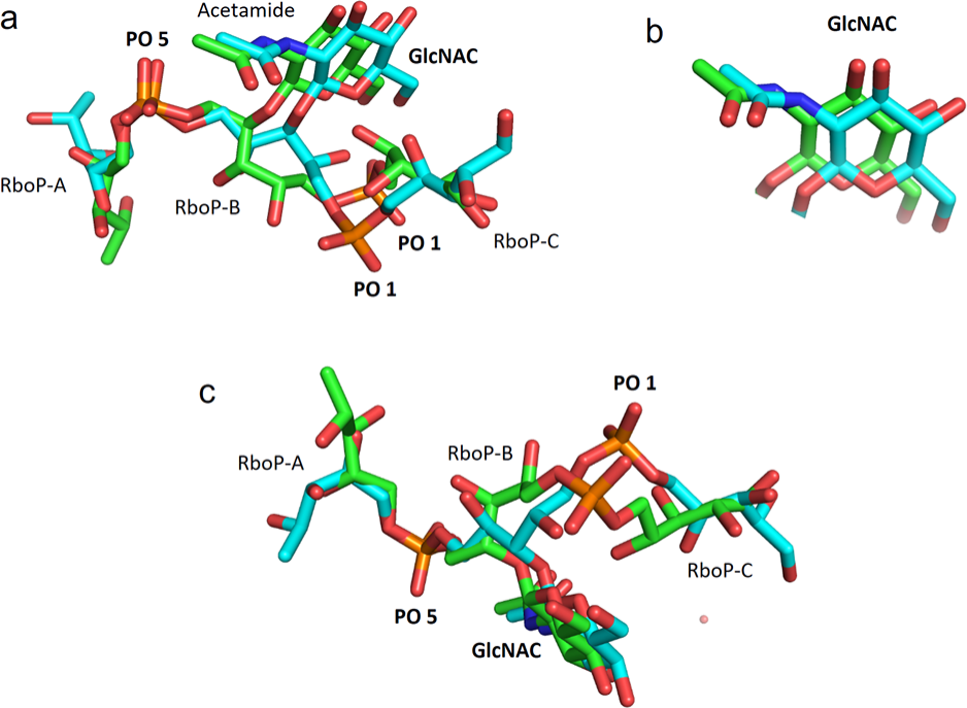
Overlap of the ligands (β-1,4)-WTA trimer (**2**) and (β-1,3)-WTA trimer (**3**) within the binding site of IgG mAb 4497. (a) Overlap of ligands **2** and **3** within the binding site cavity of mAb IgG 4497. (b) Enlarged view of the overlap of the GlcNAc rings of ligands **2** and **3**. (c) Lateral view of the overlapping ligands **1** and **2**. Ligands **2** and **3** are shown in green and cyan respectively.

Indeed, the GlcNAc residue, both phosphate groups (PO 1 and PO 5), Rbo-C and Rbo-A units participated in the interaction (Figure 9). Residue N53 is farther away from GlcNac C6-OH (4.1 Å), and no obvious H-bond formation is observed, as in the case of S31 of the heavy chain, which is farther away from PO1 and no obvious H-bond formation is observed. Residues R31 and R34 of the light chain can form H-bonds directly with phosphate group 5 and R34 also with phosphate group 1. In a similar way to ligand **2**, residue S33 stablishes a H-bond with a hydroxyl group from Rbo-A. Finally, unlike the case for ligand **2**, residue D100 is close enough to Rbo-C4 to form a H-bond at 3.3 Å, thus stabilizing the flexible ribitol unit C and assisting the accommodation of the ligand within the binding site cavity. When superimposing the crystallographic structures of mAb IgG 4497 in complexes with ligands **2** and **3**, we observed the same orientation of the GlcNAc residue and nearly the same orientation of both phosphate groups (Figure 10a–c). Finally, even though the Rbo units A and C are quite flexible, they were sufficiently well defined in both crystal structures and interactions with hydroxyl groups of the Rbo units could be observed.

#### mAb 4497–(β1,3)-GlcNAc WTA-Trimer **3**: NMR and MD Data

**Figure 11:**
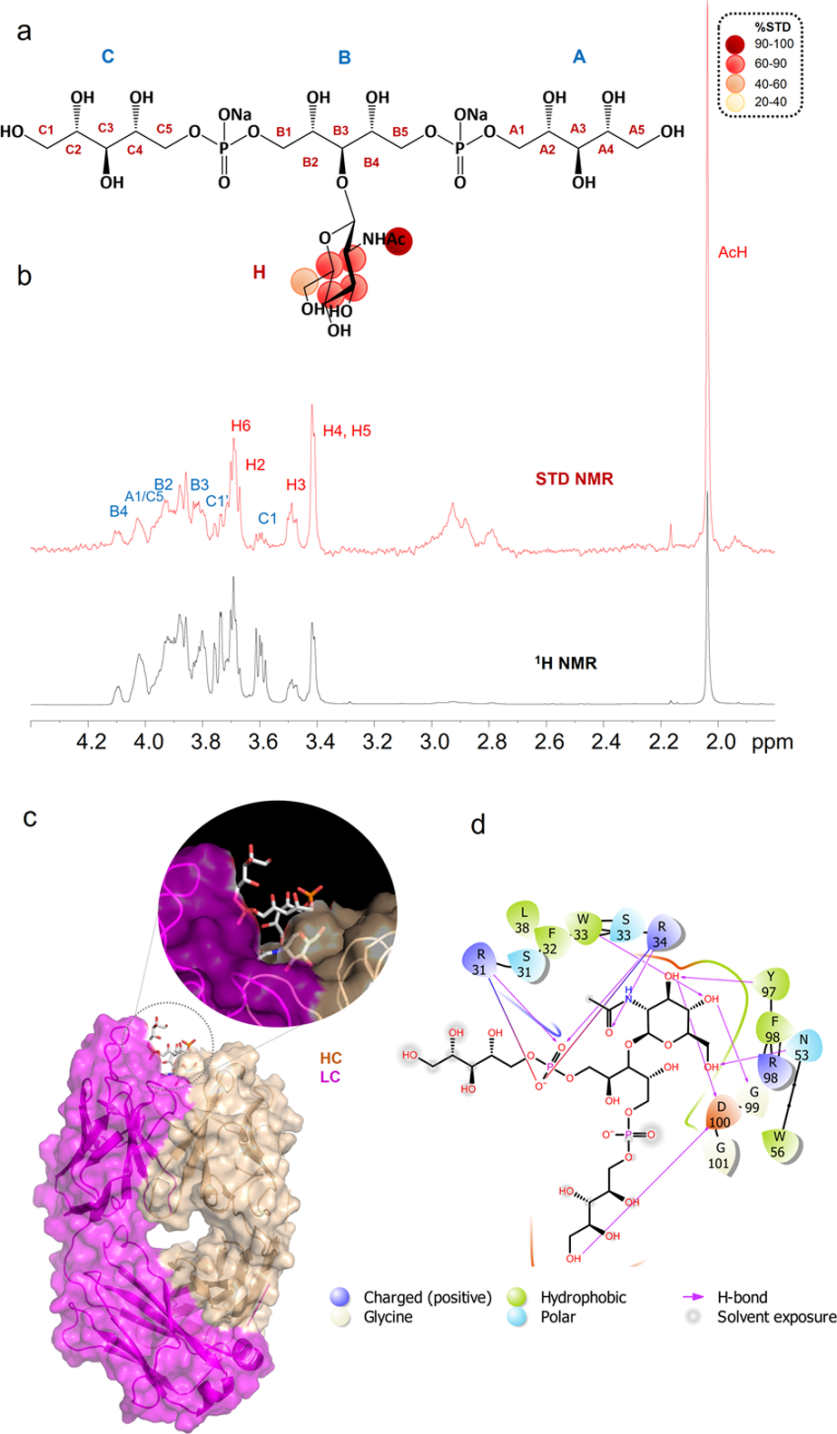
STD NMR and MD analysis of mAb 4497 and WTA-trimer **3** binding. (a) Epitope map of **3** interacting with mAb 4497 (only protons exhibiting %STD values above 30% are shown). (b) STD-NMR spectrum (red) of mAb 4497–**3** and the unsaturated reference spectrum (blue). (c) 3D view of the mAb 4461–**3** complex obtained from an MD simulation. (d) Description of the interactions between mAb 4497 and **3** resulting from a representative pose obtained by an MD simulation: solid arrows represent hydrogen bonds with functional groups of the amino acids of the antibody; the other residues in the binding pocket participate in polar and hydrophobic interactions.

The STD NMR analysis of mAb 4497 in complex with WTA-trimer **3** (Figure 11a,b and Table S4) confirmed similar binding modes for ligands **2** and **3**. As observed for ligand **2** and further supported by computational studies, we revealed the strong involvement in the binding of the sugar residue, which dropped in between the heavy- and light-chain CDRs, and a smaller contribution to the interaction from the RboP backbone.

Figure 11c,d reports one of the most representative poses resulting from the cluster analysis of the MD simulation carried out on the X-ray-derived structure of **3** in complex with mAb 4497. In agreement with the crystallographic results, the GlcNAc residue was anchored into the binding site, forming an extended network of polar and hydrophobic interactions with the antibody. Despite the flexibility of the Rbo arms along the trajectory, a partial involvement of the RboP chains in binding could be observed. In particular, the protons of the hydroxyl groups at positions 1–3 of the RboP-C unit were all interacting with D100, while the RboP-A arm was more solvent exposed. This was in accordance with the corresponding slight STD effects observed in the NMR analysis.

Overall, our results showed that the binding interactions of mAb 4497 with ligands **2** and **3** are highly concordant, with six principal residues (W33, G99, D100, Y97, S31, and N53) interacting directly with the β-GlcNAc sugar. The interaction between the antibody and the phosphate groups seemed to be crucial for stabilizing the binding in both complexes. Moreover, interactions with the RboP backbone were observed, indicating its importance in the binding.

## Discussion

We have described the structural features necessary for the recognition of *S. aureus* WTAs by WTA-specific mAbs. We exploited well-defined synthetic WTA fragments, (α-1,4)-, (β-1,4)-, and (β-1,3)-GlcNAc-WTA-trimers **1**–**3** (Figure 2), respectively, to characterize their interaction with WTA-targeting antibodies. By using an integrated approach that combines microarray, synthetic chemistry, X-ray crystallography, NMR spectroscopy, and computational studies, we showed that *S. aureus* WTA glycoforms bind the mAbs 4461 and 4497 in a cavity formed between the CDRs of the heavy and light chains. We defined that all the structural features in the WTA fragments play a role in the interaction. The GlcNAc moiety is a prime recognition element, being inserted in the antibody binding pocket, and the RboP residue to which the GlcNAc is appended and both phosphates interacted with the antibody surface participating in the binding.

Specifically, we revealed that the (α-1,4)-GlcNAc specific mAb 4461 displayed an “end-on” insertion binding mode with the antigen-binding-site amino acids forming numerous contacts with the α-GlcNAc residue and interactions with the RboP backbone. The phosphate groups interacted with the antibody through polar interactions and H-bonds. Differences in the binding of our ligands and those previously reported were observed. The structures that were previously used deviate from our structures and the naturally occurring WTA, in that they contain a single phosphate monoester as opposed to the natural phosphodiesters. We also demonstrated that the internal RboP monomer carrying the α-GlcNAc moiety is more involved in the interaction with mAb 4461 in comparison to the terminal RboP units that instead protruded away from the binding site.

Thus, the set of mAbs and well-defined ligands allowed us to unveil the binding modes of α- and β-WTA fragments in the interaction with mAbs 4461 and 4497. This provides an atomic explanation for the observed binding differences between internal and terminal α-GlcNAc-modified WTAs as well as between RboP- and GroP-based teichoic acids. Finally, our results provided insight into the cross-reactivity of 4497 for β-1,3-/β-1,4-GlcNAc-modified WTAs. We indeed showed that ligands **2** and **3** bound to mAb 4497 in a very similar manner, interacting not only with the antibody via a GlcNAc residue but also with the flexible RboP chains which permit positioning of both glycans in the binding pocket of this mAb. The results presented here have revealed the GlcNAc-RboP trimers to be the minimal synthetic epitopes that present all structural features required for binding, indicating that these can be attractive components in the generation of future synthetic antistaph conjugate vaccines. However, more research is needed to investigate the role of d-alanylation of wall teichoic acids in antibody recognition and interaction. As was mentioned above, the WTA RboP backbone can be further decorated, depending on the cellular environment conditions,^22^ with positively charged d-alanine esters, usually attached at position 2 of ribitol. d-Alanylation imparts additional WTA structural diversity and further tunes the WTA function modulating the polymer surface charge.^23^d-Ala residues indeed have the ability to mask the negatively charged sites on WTAs, increasing the bacterial resistance against host defense, antibiotics, and cationic antimicrobial peptides.^24^ Thus, d-alanylation could alter antibody recognition of teichoic acids, by direct means and/or through altering the polymer flexibility. To verify this hypothesis, ongoing efforts are aimed at the synthesis of appropriate WTA constructs, bearing the labile d-Ala esters, that will serve as tools to shed light on the role of d-alanylation in WTA recognition by monoclonal antibodies. Together with the outcomes presented here, these structural results may guide the engineering of mAbs to shape their interactions with different WTA glycotypes. This will open new avenues for the development of potential therapeutic approaches against (methicillin-resistant) *S. aureus*.

## Supporting Information Available

Additional figures and experimental procedures employed for the production of antibodies and ligands, glycan microarray, NM and X-ray experiment (PDF)

## Abbreviations

MRSA: methicillin-resistant S. aureus
WTA: wall teichoic acid
RboP: ribitolphosphate
mAb: monoclonal antibody
GroP: glycerolphosphate
LTA: lipoteichoic acid
CDR: complementarity-determining region
NMR: nuclear magnetic resonance
MD: molecular dynamics

## References

[ref1] Krismer B.; Weidenmaier C.; Zipperer A.; Peschel A. The commensal lifestyle of Staphylococcus aureus and its interactions with the nasal microbiota.. Nat. Rev. Microbiol 2017, 15 (11), 675–687. 10.1038/nrmicro.2017.104. 29021598

[ref2] Lehar S. M.; Pillow T.; Xu M.; Staben L.; Kajihara K. K.; Vandlen R.; DePalatis L.; Raab H.; Hazenbos W. L.; Hiroshi Morisaki J.; et al. S. Novel antibody-antibiotic conjugate eliminates intracellular S. aureus.. Nature 2015, 527 (7578), 323–328. 10.1038/nature16057. 26536114

[ref3] Tacconelli E.; Carrara E.; Savoldi A.; Harbarth S.; Mendelson M.; Monnet D. L.; Pulcini C.; Kahlmeter G.; Kluytmans J.; Carmeli Y.; et al. Discovery, Research, and Development of New Antibiotics: The WHO Priority List of Antibiotic-Resistant Bacteria and Tuberculosis.. Lancet Infectious Diseases 2018, 18 (3), 318–327. 10.1016/S1473-3099(17)30753-3. 29276051

[ref4] Weidenmaier C.; Peschel A. Teichoic acids and related cell-wall glycopolymers in Gram-positive physiology and host interactions.. A. Nat. Rev. Microbiol 2008, 6 (4), 276–287. 10.1038/nrmicro1861. 18327271

[ref5] Kurokawa K.; Takahashi K.; Lee B. L. The staphylococcal surface-glycopolymer wall teichoic acid (WTA) is crucial for complement activation and immunological defense against Staphylococcus aureus infection.. Immunobiology 2016, 221 (10), 1091–1101. 10.1016/j.imbio.2016.06.003. 27424796

[ref6] Boldock E.; Surewaard B. G. J.; Shamarina D.; Na M.; Fei Y.; Ali A.; Williams A.; Pollitt E. J. G.; Szkuta P.; Morris P.; et al. Human Skin Commensals Augment Staphylococcus Aureus Pathogenesis.. Nat. Microbiol 2018, 3 (8), 881–890. 10.1038/s41564-018-0198-3. 30013237 PMC6207346

[ref7] Swoboda J. G.; Campbell J.; Meredith T. C.; Walker S. Wall Teichoic Acid Function, Biosynthesis, and Inhibition.. Chem. Eur. J. of Chem. Bio. 2010, 11 (1), 35–45. 10.1002/cbic.200900557. PMC2798926 19899094

[ref8] Mistretta N.; Brossaud M.; Telles F.; Sanchez V.; Talaga P.; Rokbi B. Glycosylation of Staphylococcus aureus cell wall teichoic acid is influenced by environmental conditions.. Sci. Rep 2019, 9 (1), 3212 10.1038/s41598-019-39929-1. 30824758 PMC6397182

[ref9] Winstel V.; Xia G.; Peschel A. Pathways and roles of wall teichoic acid glycosylation in Staphylococcus aureus.. International Journal of Medical Microbiology 2014, 304 (3–4), 215–221. 10.1016/j.ijmm.2013.10.009. 24365646

[ref10] Gerlach D.; Guo Y.; De Castro C.; Kim S.-H.; Schlatterer K.; Xu F.-F.; Pereira C.; Seeberger P. H.; Ali S.; Codée J.; et al. Methicillin-resistant Staphylococcus aureus alters cell wall glycosylation to evade immunity. Nature 2018, 563 (7733), 705–709. 10.1038/s41586-018-0730-x. 30464342

[ref11] van Dalen R.; Peschel A.; van Sorge N. M. Wall Teichoic Acid in Staphylococcus aureus Host Interaction.. Trends in Microbiology 2020, 28 (12), 985–998. 10.1016/j.tim.2020.05.017. 32540314

[ref12] Takahashi K.; Kurokawa K.; Moyo P.; Jung D.-J.; An J.-H.; Chigweshe L.; Paul E.; Lee B. L. Intradermal Immunization with Wall Teichoic Acid (WTA) Elicits and Augments an Anti-WTA IgG Response that Protects Mice from Methicillin-Resistant Staphylococcus aureus Infection Independent of Mannose-Binding Lectin Status.. PLoS One 2013, 8 (8), e69739 10.1371/journal.pone.0069739. 23936347 PMC3732247

[ref13] Raafat D.; Otto M.; Reppschläger K.; Iqbal J.; Holtfreter S. Fighting Staphylococcus aureus Biofilms with Monoclonal Antibodies.. Trends in Microbiology 2019, 27 (4), 303–322. 10.1016/j.tim.2018.12.009. 30665698 PMC6420399

[ref14] Fong R.; Kajihara K.; Chen M.; Hotzel I.; Mariathasan S.; Hazenbos W. L. W.; Lupardus P. J. Structural investigation of human S. aureus-targeting antibodies that bind wall teichoic acid.. MAbs 2018, 10 (7), 979–991. 10.1080/19420862.2018.1501252. 30102105 PMC6204806

[ref15] Ali S.; Hendriks A.; Dalen R.; Bruyning T.; Meeuwenoord N.; Overkleeft H. S.; Filippov D. V.; Marel G. A.; Sorge N. M.; Codée J. D. C. (Automated) Synthesis of Well-defined Staphylococcus Aureus Wall Teichoic Acid Fragments.. Chem. - Eur. J. 2021, 27 (40), 10461–10469. 10.1002/chem.202101242. 33991006 PMC8361686

[ref16] Brown E.; Darwish M.; Flygare J.; Hazenbos W.; Lee B.-C.; Lehar S. M.; Mariathasan S.; Morisaki J. H.; Pillow T. H.; Staben L.; Vandlen R.; Koefoed K.; Strandh M.; Andersen P. S. Anti-Wall Teichoic Antibodies and Conjugates. Patent WO 2014/194247 A1, 2014.

[ref17] van Dalen R.; Molendijk M. M.; Ali S.; van Kessel K. P. M.; Aerts P.; van Strijp J. A. G.; de Haas C. J. C.; Codée J. D. C.; van Sorge N. M. Do not discard Staphylococcus aureus WTA as a vaccine antigen.. Nature 2019, 572 (7767), E1–E2. 10.1038/s41586-019-1416-8. 31367020

[ref18] van der Es D.; Berni F.; Hogendorf W. F. J.; Meeuwenoord N.; Laverde D.; van Diepen A.; Overkleeft H. S.; Filippov D. V.; Hokke C. H.; Huebner J.; et al. Streamlined Synthesis and Evaluation of Teichoic Acid Fragments.. Chem. - Eur. J. 2018, 24 (16), 4014–4018. 10.1002/chem.201800153. 29389054 PMC5887911

[ref19] Berni F.; Wang L.; Kalfopoulou E.; Nguyen D. L.; van der Es D.; Huebner J.; Overkleeft H. S.; Hokke C. H.; van der Marel G. A.; van Diepen A.; et al. Generation of Glucosylated Sn-1-Glycerolphosphate Teichoic Acids: Glycerol Stereochemistry Affects Synthesis and Antibody Interaction.. RSC Chem. Biol. 2021, 2 (1), 187–191. 10.1039/D0CB00206B. 34458781 PMC8341164

[ref20] Berni F.; Kalfopoulou E.; Gimeno Cardells A. M.; Carboni F.; van der Es D.; Romero-Saavedra F.; Laverde D.; Miklic K.; Malic S.; Rovis T. L.; et al. Epitope Recognition of a Monoclonal Antibody Raised against a Synthetic Glycerol Phosphate Based Teichoic Acid.. ACS Chem. Biol. 2021, 16 (8), 1344–1349. 10.1021/acschembio.1c00422. 34255482 PMC8389533

[ref21] Di Carluccio C.; Forgione M. C.; Martini S.; Berti F.; Molinaro A.; Marchetti R.; Silipo A. Investigation of protein-ligand complexes by ligand-based NMR methods.. Carb res 2021, 503, 108313 10.1016/j.carres.2021.108313. 33865181

[ref22] Brown S.; Santa Maria Jr J. P.; Walker S. Wall Teichoic Acids of Gram-Positive Bacteria.. Annu. Rev. Microbiol. 2013, 67, 67 10.1146/annurev-micro-092412-155620. PMC3883102 24024634

[ref23] Peschel A.; Otto M.; Jack R. W.; Kalbacher H.; Jung G.; Götz F. Inactivation of the dlt operon in Staphy-lococcus aureus confers sensitivity to defensins, protegrins, and other antimicrobial peptides.. J. Biol. Chem. 1999, 274, 8405–8410. 10.1074/jbc.274.13.8405. 10085071

[ref24] Edgar R. J.; van Hensbergen V. P.; Ruda A.; Turner A. G.; Deng P.; Le Breton Y.; El-Sayed N. M.; Be-lew A. T.; McIver K. S.; McEwan A. G.; et al. Discovery of glycerol phosphate modification on streptococcal rhamnose polysaccharides.. Nat. Chem. Biol. 2019, 15, 463–471. 10.1038/s41589-019-0251-4. 30936502 PMC6470023

