# Supplementary material for: Antibody Recognition of Different Staphylococcus aureus Wall Teichoic Acid Glycoforms
Source: ACS Cent Sci. 2022 Aug 17;8(10):1383–92. doi: 10.1021/acscentsci.2c00125 (PMC9615122; doi:10.1021/acscentsci.2c00125)
Supplement: Supplementary file 1 — oc2c00125_si_001.pdf [file oc2c00125_si_001.pdf]

Supporting Information

Antibody recognition of different *Staphylococcus aureus* wall teichoic acid glycoforms.

Cristina Di Carluccio,[a] Pablo Soriano-Maldonado,[b] Francesca Berni,[c] Carla J.C. de Haas,[d] A. Robin Temming,[e] Astrid Hendriks,[e] Sara Ali,[c] Antonio Molinaro,[a] Alba Silipo,[a] Nina M. van Sorge,[e,f]* Mark J. van Raaij,[b*,] Jeroen D.C. Codee,[c*] Roberta Marchetti[a*]

*Corresponding authors: n.m.vansorge@amsterdamumc.nl; mjvanraaij@cnb.csic.es; jcodee@chem.leidenuniv.nl; roberta.marchetti@unina.it

[a] Department of Chemical Sciences, University of Naples Federico II, Via Cinthia 4, 80126, Naples, Italy.

[b] Departamento de Estructura de Macromoléculas, Centro Nacional de Biotecnología, Consejo Superior de Investigaciones Científicas (CNB-CSIC) Calle Darwin 3, 28049 Madrid, Spain

[c] Leiden Institute of Chemistry, Leiden University, Einsteinweg 55, 2333 CC Leiden, the Netherlands

[d] Medical Microbiology, UMC Utrecht, Utrecht University, Utrecht, The Netherlands

[e] Department of Medical Microbiology and Infection Prevention, Amsterdam UMC, University of Amsterdam, 1105 AZ, Amsterdam, the Netherlands

[f] Netherlands Reference Laboratory for Bacterial Meningitis, Amsterdam UMC, location AMC, 1105 AZ, Amsterdam, The Netherlands

Table of Contents

Table S1 Crystallographic data, refinement and model statistics. pag S3

Table S2 ^{1}H and ^{13}C chemical shift (ppm) values of trimer 1 at 298 K. pag S4

Table S3 ^{1}H and ^{13}C chemical shift (ppm) values of trimer 2 at 298 K. pag S4

Table S4 ^{1}H and ^{13}C chemical shift (ppm) values of trimer 3 at 298 K. pag S4

Table S5 Summary of binding interactions by X-ray. pag S5

Figure S1 mAb 4461/**1** 2D diagram of the interactions. pag S6

Experimental section pag S7-S9

References pag S9-S10

Table S1. Crystallographic data, refinement and model statistics.

**Data collection**	**Fab 4461-compound 1**	**Fab 4497-compound 2**	**Fab 4497-compound 3**
Wavelength (Å)	0.97926	0.97933	0.97926
Space group	$P2_1$	$P2_12_12_1$	$P2_12_12_1$
Cell edges (a, b, c, Å) / angle (β, º)	58.19, 65.45, 74.34 / 112.9	64.39, 113.37, 154.56 / 90.0	64.03, 112.92, 154.99 / 90.0
Resolution range[a] (Å)	53.51-1.45 (1.53-1.45)	91.41-1.65 (1.74-1.65)	47.0-1.84 (1.90-1.84)
Number of unique reflections	87588 (10122)	127285 (19760)	97934 (9538)
Completeness (%)	96.6 (80.6)	92.9 (100.0)	100.0 (100.0)
Multiplicity	3.1 (2.5)	6.5 (6.5)	6.6 (6.5)
CC1/2[b]	0.997 (0.608)	0.998 (0.661)	0.999 (0.601)
Rmeas[c]	0.039 (0.593)	0.092 (1.248)	0.100 (2.124)
<I/s(I)>	16.4 (1.5)	12.0 (1.5)	11.6 (0.9)
Wilson B (Å^2)	21.8	24.4	34.1
**Refinement statistics**			
Resolution range[a] (Å)	53.5 - 1.45 (1.53-1.45)	90.0 - 1.65 (1.74-1.65)	46.0-1.84 (1.94-1.84)
Reflections used in refinement	83262 (10122)	127097 (18773)	98170 (13327)
Reflections used for R-free	4368 (529)	6287 (962)	4988 (680)
R-factor[d]	0.174 (0.335)	0.175 (0.292)	0.188 (0.342)
R-free[e]	0.200 (0.342)	0.202 (0.299)	0.218 (0.342)
**Model statistics**			
Atoms (protein / WTA ligand / CAPSO / PEG / glycerol / sulfate / Cl$^-$ / water)	3323 / 45 / 15 / 28 / 0 / 0 / 0 / 424	6669 / 96 / 0 / 7 / 12 / 10 / 0 / 829	6632 / 91 / 0 / 14 / 6 / 25 / 1 / 507
Average temperature factor (Å^2, protein / WTA ligand / PEG / glycerol / sulfate / Cl$^-$ / water)	23.8 / 36.0 / 29.8 / 49.0 / - / - / - / 33.7	26.8 / 32.2 / - / 68.3 / 44.3 / 54.9 / - / 34.8	39.9 / 65.7 / - / 81.0 / 73.9 / 88.8 / 36.8 / 43.8
RMSD[f] (bonds, Å / angles, º)	0.009 / 1.5	0.010 / 1.5	0.009 / 1.5
Ramachandran plot[g] (% favoured / allowed / disallowed)	97.9 / 2.1 / 0.0	97.9 / 2.1 / 0.0	97.9 / 1.9 / 0.2
Clash score / percentile[h]	3.34 / 97th	2.25 / 99th	2.63 / 99th
Molprobity score / percentile[i]	1.13 / 98th	1.00 / 100th	1.07 / 100th
PDB code	7QXC	7QXD	7QXE

[a]The values in parenthesis correspond to the high-resolution shell

[b]Correlation coefficient between intensity estimates from half data sets[1]

[c]Rmeas is a multiplicity-independent data quality indicator calculated according to Diederichs and Karplus (1997).[2]

[d]R = Shkl|Fobs(hkl) – Fcalc(hkl)| / Shkl |Fobs(hkl)|

[e]The R-free is calculated as for R, but from a small subset of reflections excluded from refinement (and from calculation of the R-factor).

[f]Calculated by REFMAC5 [3]

[g]Ramachandran plot from MOLPROBITY [4]

[h]The clash score is the number of serious clashes per 1000 atoms, while the 100th percentile is the best among structures of comparable resolution and the 0th percentile is the worst.

[i]The Molprobity score combines the clash score, rotamer and Ramachandran evaluations into a single score, normalized to be on the same scale as the X-ray resolution (http://molprobity.biochem.duke.edu/). [17]

Table S2. ^{1}H **and** ^{13}C **chemical shift (ppm) values of trimer 1 at 298 K.**

Unit	1	2	3	4	5	6	Ac
**GlcNAc H residue**	4.998	3.855	3.758	3.432	3.957	3.829/3.726	2.006
	96.47	53.86	70.93	70.05	71.90	60.64	22.06
**Rbo chain A residue**	3.994/3.899	3.845	3.683	3.796	3.745/3.589		
	66.57	70.05	71.76	72.08	62.41		
**Rbo chain B residue**	4.057/4.012	3.872	3.855	4.003	4.057/4.012		
	64.47	71.06	70.96	77.71	64.47		
**Rbo chain C residue**	3.745/3.589	3.796	3.683	3.845	3.994/3.899		
	62.41	72.08	71.76	70.05	66.57		

Table S3. ^{1}H **and** ^{13}C **chemical shift (ppm) values of trimer 2 at 298 K.**

Unit	1	2	3	4	5	6	Ac
**GlcNAc H residue**	4.677	3.687	3.499	3.403	3.401	3.697/3.871	2.027
	101.61	55.62	73.83	69.64	75.72	60.59	22.45
**Rbo chain A residue**	3.911/4.009	3.860	3.679	3.797	3.593/3.743		
	66.58	70.1	71.64	71.99	62.25		
**Rbo chain B residue**	3.891/4.064	3.870	3.871	4.096	3.891/4.064		
	64.99	70.89	70.14	79.79	64.99		
**Rbo chain C residue**	3.593/3.743	3.797	3.679	3.860	3.911/4.009		
	62.25	71.99	71.64	70.1	66.58		

Table S4. ^{1}H **and** ^{13}C **chemical shift (ppm) values of trimer 3 at 298 K.**

Unit	1	2	3	4	5	6	Ac
**GlcNAc H residue**	4.558	3.661	3.457	3.390	3.384	3.676/3.840	2.010
	101.89	55.65	73.96	69.75	75.68	60.52	22.29
**Rbo chain A residue**	3.883/3.995	3.852	3.670	3.774	3.569/3.720		
	66.43	71.15	72.20	72.05	62.30		
**Rbo chain B residue**	3.830/3.934	3.910	3.797	4.074	3.830/3.934		
	66.40	69.60	80.70	71.0	66.40		
**Rbo chain C residue**	3.569/3.720	3.774	3.670	3.852	3.883/3.995		
	62.30	72.05	72.20	71.15	66.43		

Table S5. Summary of binding interactions by X-ray

	GlcNAc	PO 1	PO 5	Rbo-A	Rbo-B	Rbo-C
**4461 α(1,4)**						
	Y97 - C_3OH - 2.7Å, NHAc - 3.5 Å<br>Y98 - NHAc - 2.9 Å<br>S100 - C_4OH - 2.5 Å<br>Y33 - C_6OH - 3.6 Å	Y98 - 2.8 Å<br>S31 - 2.8 Å	S100 - 2.7 Å	S100 - Rbo-A2 - 3 Å	Y98 - Rbo-B3 - 2.6 Å	
**4497 β(1,4)**						
	W33 - stacking, C_6OH - 3 Å<br>Y97 - C_3OH - 2.6 Å, NHAc - 3.7 Å<br>G99 - C_4OH - 2.7 Å<br>D100 - C_3OH - 2.5 Å<br>N53 - C_6OH - 3.2 Å<br>S31 - C_6OH - 3.6 Å<br>R34 - Acetamide - 3 Å	S31 - 2.7 Å Wm	R32 - 2.9 Å & 3.5 Å<br>R34 - 2.9 Å & 3.1 Å	R34 - Rbo-A3 - 3.8 Å Wm<br>S33 - Rbo-A3 - 2.8 Å	R34 - Rbo-B3 - 3.8 Å Wm	D100 - Rbo-C4 - 3.8 Å<br>R34 - Rbo-C4 - 2.8 Å Wm
**4497 β(1,3)**						
	W33 - stacking, C_6OH - 2.8 Å<br>Y97 - C_3OH - 2.6 Å, NHAc - 3.6 Å<br>G99 - C_4OH - 2.6 Å<br>D100 - C_3OH - 2.5 Å<br>N53 - C_6OH - 4.1 Å*<br>S31 - 3.6 Å<br>R34 - Acetamide – 3.1 Å	R34 - 3.2 Å & 2.8 Å Wm	R32 - 3.1 Å & 3.2 Å<br>R34 - 2.9 Å	S33 - Rbo-A2 - 2.9 Å		D100 – Rbo-C4 - 3.3 Å

Wm: The H-bond is formed through a water molecule.
*: no H-bond is expected due to the high distance between both residues.

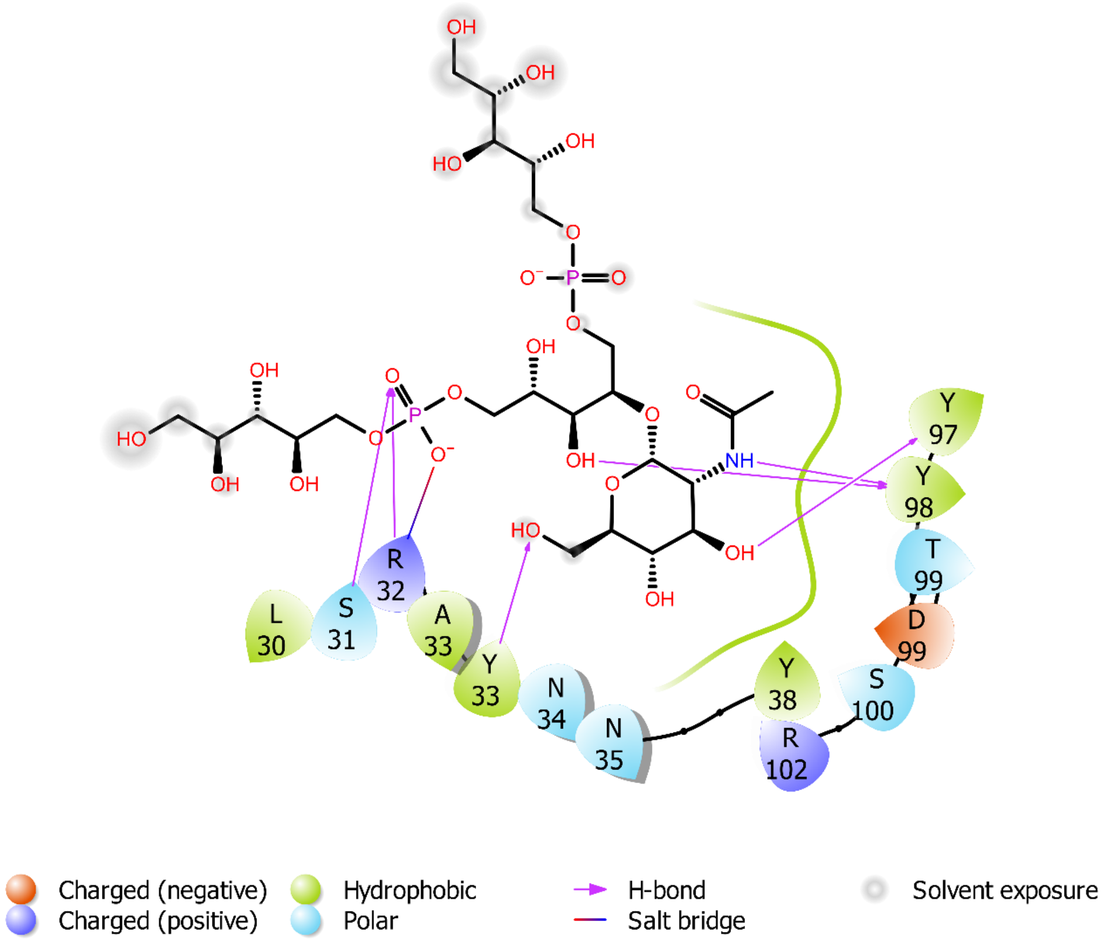

Figure S1. The 2D diagram of the interactions between mAb 4461 and **1** resulting from another representative pose obtained by MD is shown. The H-bonds between S31 e R32 with PO1 and Y33 with OH at position 6 of GlcNAc are visible.

Experimental section

Production of anti-WTA monoclonal antibodies and synthetic WTA fragments

Anti-WTA clones 4497 and 4461 were produced as IgG1 and Fab fragments as described previously.[5,6] Synthetic WTA fragments were generated as already described. [4]

Microarray

The amino-spacer equipped synthetic fragments were dissolved in spotting buffer (Nexterion Spot, Schott Nexterion) with 10% DMSO in 384-wells V-bottom plates (Genetix, New Milton, UK). The fragments were printed in three final concentrations (30 µM, 10 µM and 3 µM) in triplicate on epoxysilane-coated glass slides (Slide E, Schott, Nexterion) by contact printing using the Omnigrid 100 microarrayer (Genomic Solutions, Ann Arbor, MI) equipped with SMP3 pins with uptake channels that deposit 0.7 nl at each contact. The slides were rested in a high humidity chamber for 18 hours and were stored in the dark until used. The slides were washed with PBS (3x) and subsequently all unreacted sites on the arrays were blocked by shaking the slides for 1 hour with ethanolamine (0.10 ml, 0.05 M in PBS containing 20 mg/ml of BSA). The slides were flushed with PBS containing 5% of Tween® 20 and then PBS and finally rinsed with PBS containing 1% of Tween® 20. After removal of the PBS containing 1% of Tween® 20, the arrays were shaken with designated monoclonal (4497 or 4461) at 1 µg/ml, 0.5 µg/ml and 0,25 µg/ml (0.10ml, diluted with PBS containing 1% of Tween® 20 and 10 mg/ml of BSA) for 60 minutes. The slides were flushed with PBS containing 5% of Tween® 20 and PBS and rinsed with PBS containing 1% of Tween® 20 subsequently. After removal of the PBS containing 1% of Tween® 20, the arrays were shaken with goat anti-human IgG secondary antibody, Alexa Fluor® 488 conjugate (Invitrogen, A-11013), (0.10 ml, 0.5 µg/ml final dilution in PBS containing 1% of Tween® 20 and 10 mg/ml of BSA) for 30 minutes in the dark. The slides were flushed with PBS containing 5% of Tween® 20, PBS and MilliQ subsequently. The slides were dried by centrifugation and were analyzed on fluorescence on 532 nm and 635 nm using a G2565BA scanner. Data and image analyses were performed with GenePix Pro 7.0 software (Molecular Devices, Sunnyvale, CA, USA) as described previously.[7] Fluorescence intensities were quantified and corrected for background/non-specific antibody adhesion by subtracting the fluorescence at blank spots, where spotting buffer was printed without GTA fragment. The average of the triplicate spots was normalized to the highest intensity on the array and visualized in bar graphs using GraphPad Prism 8.4.3.

X-ray crystallography

*Crystallization of Fab4461 with **1** and Fab4497 with **2** and **3***

Fab4461 was concentrated to 12 mg/ml in 10 mM Tris-HCl pH 8.5, 0.25 M sodium chloride using an Amicon Ultra 10K molecular weight cut-off centrifugal filter device (Merck Millipore, Billerica, Massachusetts, USA). The final protein concentration was determined by UV spectroscopy (Nanodrop, Thermo Scientific, Wilmington, Delaware, USA), calculating the molar extinction coefficient at 280 nm from the amino acid composition, and then stored temporarily at 4 ºC prior to use in crystallization trials. Crystallization trials were performed by sitting-drop vapour diffusion at 20 ºC in MRC crystallization plates (Molecular Dimensions, Newmarket, Suffolk, England). Crystals were obtained in a drop containing 12.9% (w/v) polyethylene glycol 20,000 150 mM 3-(cyclohexylamino)-2-hydroxy-1-propanesulfonic acid-NaOH pH 9.5, 6% (v/v) polyethylene glycol 300 and were soaked into reservoir solution containing ligand **1** at a concentration of around 1 mM.

Fab4497 was concentrated to 14 mg/ml in 10 mM 2-(N-morpholino)ethanesulfonic acid-NaOH pH 6.2, 0.25 M sodium chloride and crystallised as above. Crystals were obtained in a drop containing 20% (w/v) poly-ethylene glycol 4000, 10% (v/v) glycerol, 200 mM magnesium sulfate and soaked into reservoir solution containing ligand **2**, cryo-protected and flash-cooled as above. Crystals were also obtained in a drop containing 20% (w/v) poly-ethylene glycol 4000, 0.1 M Tris-HCl pH 8.5, 0.2 M lithium sulfate and soaked into reservoir solution containing ligand **3**. All crystals were transferred to a cryoprotectant solution consisting of the reservoir/ligand solution but including 20% (v/v) glycerol, mounted in a LithoLoop (Molecular Dimensions, Newmarket, England) and flash-cooled in liquid nitrogen.

Crystallographic data collection, processing, structure solution and refinement.

Crystallographic diffraction data for Fabs 4497 and 4461 were collected on beamline XALOC-BL13[8] of the ALBA Synchrotron Light Facility (Barcelona, Spain) using a Pilatus 6M pixel detector (Dectris Ltd, Baden, Switzerland). Crystals were kept at 100 K during data collection. Reflections were integrated with the programme XDS and reduced using POINTLESS,[9] AIMLESS,[10] and TRUNCATE,[11] all integrated in the Collaborative Computational Project Number 4 (CCP4).[12] For data statistics, see Table S1.

The structure of Fab 4461 was solved by molecular replacement using PDB entry 5I1D as a model,[13] searching for the four immunoglobulin domains separately (heavy chain constant domain and variable domain, light chain constant and variable domain). The program PHASER[14] was used. The structure was then rebuilt using ARP-WARP[15] and the correct protein sequences. The crystallographic asymmetric unit contains one ligand-bound Fab fragment. The Fab 4497 structures were solved by rigid body refinement of a previous Fab 4497 structure, PDB entry 5D6C.[16] Here, two Fabs per asymmetric unit are present, each binding a WTA ligand.

Adjustment of the models was performed with COOT [17] and refinement with REFMAC5.[3] Reflections (5%) for calculating the free R-factor were selected randomly. Validation was carried out with MOLPROBITY.[4] Structure figures were made with PYMOL (Schrödinger LLC, Cambridge MA, USA). Coordinates and structure factors have been submitted to the protein structure database, access codes are in Table S1.

NMR

Samples were prepared using a phosphate saline deuterated buffer composed of 0.01 M phosphate buffer, 0.0027 M potassium chloride and 0.137 M sodium chloride, pH 7.4 and 288 °K.

NMR experiments were recorded on a Bruker AVANCE NEO 600-MHz equipped with a cryo probe and calibrated with [D_4](trimethylsilyl)propionic acid, sodium salt (TSP, 10 µM) as an internal reference. The analyses were performed with TOPSPIN 3.2 software.

The punctual assignment of the ^{1}H and ^{13}C resonances of trimers 1-3 has been performed by a combination of 1D and 2D NMR experiments (see Tables S2-S4). The homonuclear spectra, DQF-COSY, TOCSY, NOESY, ROESY experiments were performed with data sets (t1×t2) of 4096×512 points. The data matrix was zero-filled in both dimensions to give a matrix of 4 K×2 K points and was resolution-enhanced using a cosinebell function before Fourier transformation. Data sets of 2048×256 points were used for HSQC, HSQC-TOCSY and HMBC experiments, performed in the ^{1}H-detection mode by single-quantum coherence with proton decoupling in the ^{13}C domain.

STD NMR experiments were acquired with 32 k data points and zero-filled up to 64 k data points prior to processing. The protein resonances were selectively irradiated by 40 Gauss pulses with a length of 50 ms, using the off-resonance pulse frequency at 40 ppm and on-resonance pulse at 7.5 ppm. The STD NMR spectra were recorded with an excitation sculpting to suppress the water signals and without applying gradient pulses (esgp) in order to observe the anomeric proton signals. The STD spectra were acquired at saturation time of 2 s. An antibody/ligand molar ratio of 1:50 was used for all systems. STD effects were calculated by using the ratio of $(I_0 – I_{sat})/I_0$, where I_{sat} is the intensity of the STD NMR signal in the spectrum and I_0 the peak intensity of an unsaturated reference spectrum (off-resonance). The %STD were obtained considering the acetyl group of Neu5Ac as 100%.

Molecular Dynamics

Before running MD simulations of the complexes, the non-standard RboP residues were parametrized. A *mol2* file was generated in *antechamber* program and a frcmod file was created using *parmchk2* for the force field parameters of the ligand. Once RboP was parametrized, pdb files of ligands **1**,**2** and **3** were built and the prmtop and inpcrd files were generated accordingly by using tLEaP module of AMBER 18 package[18]. GLYCAM06j-1 force field was employed to represent the carbohydrate parameters.[19] The MD simulations were performed using the CUDA,[20] implementation of PMEMD in the AMBER18 software with explicit water to simulate the aqueous environment and no torsional restrictions were applied. Prior to MD, to neutralize the system, counter ions were added by using the Leap module and the hydration of the complexes was allowed by using octahedral boxes containing explicit TIP3P water molecules extending 10 Å away from any atom. A step of minimization of each complex was performed using Sander. Since all the complexes came from well-defined crystallographic structures, 100 ns MD simulations were carried out modules in AMBER18 package. The

electrostatic attractions in the system were represented using the smooth particle mesh Ewald method applying periodic boundary conditions and the grid spacing was 1 Å. Initial annealing of the system occurred steadily and lightly from 100 °K to 300 °K over 25 ps. A constant temperature of 300 °K was kept during 50 ps, with progressive energy minimizations. Coordinates from MD were collected in order to acquire 1000 frames of the progression of the dynamics. Trajectories were submitted to cluster analysis with respect to the ligand RMSD using K-mean algorithm implemented in ptraj module in AMBER18 software and visualized with VMD molecular visualization program (Roe et al., 2013). [21] The structures of the most populated cluster were considered to depict the complexes interactions. The determination of hydrogen bonds was calculated using the CPPTRAJ module, with the atoms distance cut-off set to 3 Å and the atoms angle cut-off to 135°.

References

(1) Hendriks, A.; van Dalen, R.; Ali, S.; Gerlach, D.; van der Marel, G. A.; Fuchsberger, F. F.; Aerts, P. C.; de Haas, C. J C.; Pesche, A.; Rademacher, C.; et al. Impact of Glycan Linkage to Staphylococcus aureus Wall Teichoic Acid on Langerin Recognition and Langerhans Cell Activation. *ACS Infect. Dis.* **2021**, *7*(3),624-635.

(2) Diederichs, K.; Karplus, P.A. Improved R-factors for diffraction data analysis in macromolecular crystallography.**1997**, *Nature Structural Biology* 4, 269–275.

(3) Murshudov, G. N.; Skubák, P.; Lebedev, A. A.; Pannu, N. S.; Steiner, R. A.; Nicholls, R. A.; Winn, M. D.; Long F.; Vagin, A.A. REFMAC5 for the refinement of macromolecular crystal structures *Acta. Cryst,* **2011** D67, 355-367.

(4) Williams, C. J.; Headd, J. J.; Moriarty, N. W.; Prisant, M. G.; Videau, L. L.; Deis, L. N.; Verma, V.; Keedy, D. A.; Hintze, B. J.; Chen, V. B.; et al. MolProbity: More and Better Reference Data for Improved All-Atom Structure Validation. *Protein Sci* **2018**, *27* (1), 293–315.

(5) Hendriks, A.; van Dalen, R.; Ali, S.; Gerlach, D.; van der Marel, G. A; Fuchsberger, F. F; Aerts, P. C; de Haas, C. J A. C; Peschel, A; Rademacher, C.; et al. Impact of Glycan Linkage to Staphylococcus aureus Wall Teichoic Acid on Langerin Recognition and Langerhans Cell Activation. *ACS Infect Dis*. **2021**, *7*(3),624-635.

(6) Ali, S.; Hendriks, A.; Dalen, R.; Bruyning, T.; Meeuwenoord, N.; Overkleeft, H.S.; Filippov, D.V.; Marel, G.A.; Sorge, N.M.; Codée, J.D.C. (Automated) Synthesis of Well-defined Staphylococcus Aureus Wall Teichoic Acid Fragments. *Chem. Eur. J.* **2021**, *27* (40), 10461–10469.

(7) Oyelaran, O.; Li, Q.; Farnsworth, D.; Gildersleeve, J. C.; Microarrays with Varying Carbohydrate Density Reveal Distinct Subpopulations of Serum Antibodies. *J. Prot. Res.* **2009**, 8, 3529-3538.

(8) Juanhuix, J.; Gil-Ortiz, F.; Cuní, G.; Colldelram, C.; Nicolas, J.; Lidon, J.; Boter, E.; Ruget, C.; Ferrer, S.; Benach. J. Developments in optics and performance at BL13-XALOC, the macromolecular crystallography beamline at the Alba Synchrotron. *Synchrotron Rad.* **2014**, 21, 679–689.

(9) Evans, P.R. An introduction to data reduction: space-group determination, scaling and intensity statistics. *Acta Cryst*. **2011**, D67, 282-292.

(10) Evans, P.R.; Murshudov G.N. How good are my data and what is the resolution? *Acta Cryst*., **2013**, D69, 1204-1214.

(11) French G.S., Wilson K.S. On the Treatment of Negative Intensity Observations. *Acta. Cryst*., **1978**, A34, 517.

(12) Winn M. D. *et al*. Overview of the CCP4 suite and current developments. *Acta. Cryst.,* **2011**, D67, 235-242.

(13) Teplyakov et al., Structural diversity in a human antibody germline library mA*bS*, **2016**, *8*(6), 1045–1063.

(14) McCoy, AJ.; Grosse-Kunstleve, R.W.; Adams, P.D.; Winn, M.D.; Storoni, L.C.; Read. R.J. Phaser crystallographic software *J Appl Cryst* **2007** *40*, 658-674.

(15) Langer, G.; Cohen, S.X.; Lamzin, V.S.; Perrakis. A. Automated macromolecular model building for X-ray crystallography using ARP/wARP version 7. *Nat. Protoc*., **2008**, 3, 1171-1179.

(16) Lehar, S. M.; Pillow, T.; Xu, M.; Staben, L.; Kajihara, K.K ; Vandlen, R.; DePalatis, L.; Raab, H.; Hazenbos, W.L.; Hiroshi Morisaki, J.; et al. Novel antibody-antibiotic conjugate eliminates intracellular S. aureus. *Nature* **2015**, *527* (7578), 323–328.

(17) Emsley, P.; Lohkamp, B.; Scott, W. G.; Cowtand K. Features and development of Coot. *Acta. Cryst*. **2010**, D66, 486-501

(18) Case, D.A.; et al. Amber 2021: Reference Manual **2021**, Amber 2021, University of California, San Francisco.

(19) Kirschner, K. N., Yongye, A. B., Tschampel, S. M., González-Outeiriño, J., Daniels, C. R., Foley, B. L., & Woods, R. J. GLYCAM06: A generalizable biomolecular force field. Carbohydrates *Journal of computational chemistry*, **2008,** *29*(4), 622–655.

(20) Goetz, A.W.; Williamson, M.J.; Xu, D.; Poole, D.; Le Grand, S.; Walker, R.C. Routine Microsecond Molecular Dynamics Simulations with AMBER on GPUs. 1. Generalized Born. *J. Chem. Theory Comput.* **2012**, 8, 1542-1555. Salomon-Ferrer, R.; Goetz, A.W.; Poole; D.; Le Grand, S.; Walker. R.C. Routine Microsecond Molecular Dynamics Simulations with AMBER on GPUs. 2. Explicit Solvent Particle Mesh Ewald. *J. Chem. Theory Comput.* **2013,** 9, 3878-3888.

(21) Roe, D.R.; Cheatham, T. E. PTRAJ and CPPTRAJ: Software for Processing and Analysis of Molecular Dynamics Trajectory Data *J. Chem. Theory Comput.,* **2013**, 9(7), 3084-3095.
